# *Schistosoma mansoni Sm*KI-1 serine protease inhibitor binds to elastase and impairs neutrophil function and inflammation

**DOI:** 10.1371/journal.ppat.1006870

**Published:** 2018-02-09

**Authors:** Suellen B. Morais, Barbara C. Figueiredo, Natan R. G. Assis, Debora M. Alvarenga, Mariana T. Q. de Magalhães, Rafaela S. Ferreira, Angélica T. Vieira, Gustavo B. Menezes, Sergio C. Oliveira

**Affiliations:** 1 Departamento de Bioquímica e Imunologia, Instituto de Ciências Biológicas, Universidade Federal de Minas Gerais, Belo Horizonte, Minas Gerais, Brazil; 2 Instituto Nacional de Ciência e Tecnologia em Doenças Tropicais (INCT-DT), Conselho Nacional de Desenvolvimento Científico e Tecnológico, Ministério de Ciência Tecnologia e Inovação Salvador, Bahia, Brazil; 3 Departamento de Bioquímica e Biofísica, Instituto de Ciências da Saúde, Universidade Federal da Bahia, Salvador, Bahia, Brazil; 4 Centro de Biologia Gastrointestinal, Departamento de Morfologia do Instituto de Ciências Biológicas, Universidade Federal de Minas Gerais, Belo Horizonte, Minas Gerais, Brazil; University of Medicine & Dentistry New Jersey, UNITED STATES

## Abstract

Protease inhibitors have important function during homeostasis, inflammation and tissue injury. In this study, we described the role of *Schistosoma mansoni Sm*KI-1 serine protease inhibitor in parasite development and as a molecule capable of regulating different models of inflammatory diseases. First, we determine that recombinant (r) *Sm*KI-1 and its Kunitz domain but not the C-terminal region possess inhibitory activity against trypsin and neutrophil elastase (NE). To better understand the molecular basis of NE inhibition by S*m*KI-1, molecular docking studies were also conducted. Docking results suggest a complete blockage of NE active site by *Sm*KI-1 Kunitz domain. Additionally, r*Sm*KI-1 markedly inhibited the capacity of NE to kill schistosomes. In order to further investigate the role of *Sm*KI-1 in the parasite, we designed specific siRNA to knockdown *Sm*KI-1 in *S*. *mansoni*. *SmKI-1* gene suppression in larval stage of *S*. *mansoni* robustly impact in parasite development *in vitro* and *in vivo*. To determine the ability of *Sm*KI-1 to interfere with neutrophil migration and function, we tested *Sm*KI-1 anti-inflammatory potential in different murine models of inflammatory diseases. Treatment with *Sm*KI-1 rescued acetaminophen (APAP)-mediated liver damage, with a significant reduction in both neutrophil recruitment and elastase activity. In the model of gout arthritis, this protein reduced neutrophil accumulation, IL-1β secretion, hypernociception, and overall pathological score. Finally, we demonstrated the ability of *Sm*KI-1 to inhibit early events that trigger neutrophil recruitment in pleural cavities of mice in response to carrageenan. In conclusion, *Sm*KI-1 is a key protein in *S*. *mansoni* survival and it has the ability to inhibit neutrophil function as a promising therapeutic molecule against inflammatory diseases.

## Author summary

*Schistosoma mansoni* is one of the main agents of schistosomiasis, which is the most important human helminthic infection in terms of global morbidity and mortality. Although schistosomiasis represents a major public health problem in endemic countries, evidences show that *S*. *mansoni* downregulates inflammatory responses in many diseases. Fortunately, the control of inflammatory responses is extended to pathogen-derived antigens, leading us to study one *S*. *mansoni* Kunitz type protease inhibitor (*Sm*KI-1), found in larval and adult phases of the parasite. We demonstrate that *Sm*KI-1 inhibits trypsin and neutrophil elastase (NE). Additionally, live parasites that *SmKI-1* gene has been suppressed using siRNA displayed an impaired schistosome development both *in vitro* and *in vivo*. Further, we demonstrate that *Sm*KI-1 possesses an anti-inflammatory potential in three different murine models of inflammatory diseases: acetaminophen (APAP)-mediated liver damage, gout arthritis, and pleural inflammation in response to carrageenan. In these inflammatory disease models, we evaluated *Sm*KI-1 effect on neutrophil and our results demonstrate this molecule is able to inhibit neutrophil migration and function, regulating inflammation. Thus, our data suggest that *Sm*KI-1 is a promising therapeutic molecule against inflammatory diseases.

## Introduction

Immunologic disorders are becoming increasingly prevalent in developed population, with asthma exceeding 10% of children in many countries [1], while the incidence of autoimmune diseases such as type I diabetes [2] as well as of inflammatory bowel disease (IBD)[3] continues to rise. Helminth parasites are associated with protection from inflammatory conditions in both humans and animal models [4]. One possible explanation resides that helminths drive the regulatory arm of the immune system, abrogating the ability of the host to expel the parasites, while also dampening reactivity to many inflammatory processes. Furthermore, these parasites or their products hold therapeutic potential for human inflammatory disorders.

Parasitic helminths of genus *Schistosoma*, the agents of schistosomiasis, develop the capability to live for decades in the blood vessels of human host. Schistosomiasis is the most important human helminthic infection in terms of global morbidity and mortality [5]. During infection, larval schistosomes (schistosomula) migrate to the blood vessels where they mature to adult worms and live as pairs in the mesenteric or perivesicular veins for years [6]. These parasite stages (larval and adult), besides surviving inside the host, acquire the ability to modulate human immune responses, what has always called the attention of many scientists worldwide [7, 8]. Many studies reveal that schistosomes down-regulate inflammatory responses in immune-mediated diseases [9–12]. Fortunately, the control of inflammation seems not to be strictly dependent on parasite infection, since it is extended to some pathogen-derived antigens [9, 11, 12], suggesting some schistosome molecules are useful weapons to control inflammation [13, 14].

Immunity to schistosome infection has been assigned to several immune mechanisms including parasite opsonization by specific antibodies and complement and various types of effector cells (e.g., neutrophils, macrophages, eosinophils, and others) [15, 16]. Among immune cells, neutrophils are stated as the first cell type to arrive at inflammatory sites [17]. These cells have a crucial role on innate immune response development [17] although they are also involved in host tissue damage through secretion of proteases and cytotoxic mediators [18]. In this study, we characterized biochemically *S*. *mansoni Sm*KI-1 and we showed that expression of *Sm*KI-1 is essential for parasite survival *in vitro* and *in vivo*. Additionally, we tested *Sm*KI-1 anti-inflammatory potential in different murine models of inflammatory diseases, such as acetaminophen (APAP)-induced liver inflammation, gout arthritis and carrageenan-induced pleural inflammation. Finally, we observed that *Sm*KI-1 has the ability to bind to elastase and interferes with neutrophil migration and function, reducing inflammation.

## Results

### *Schistosoma mansoni* SmKI-1 is a Kunitz type serine-protease inhibitor

**Fig 1:**
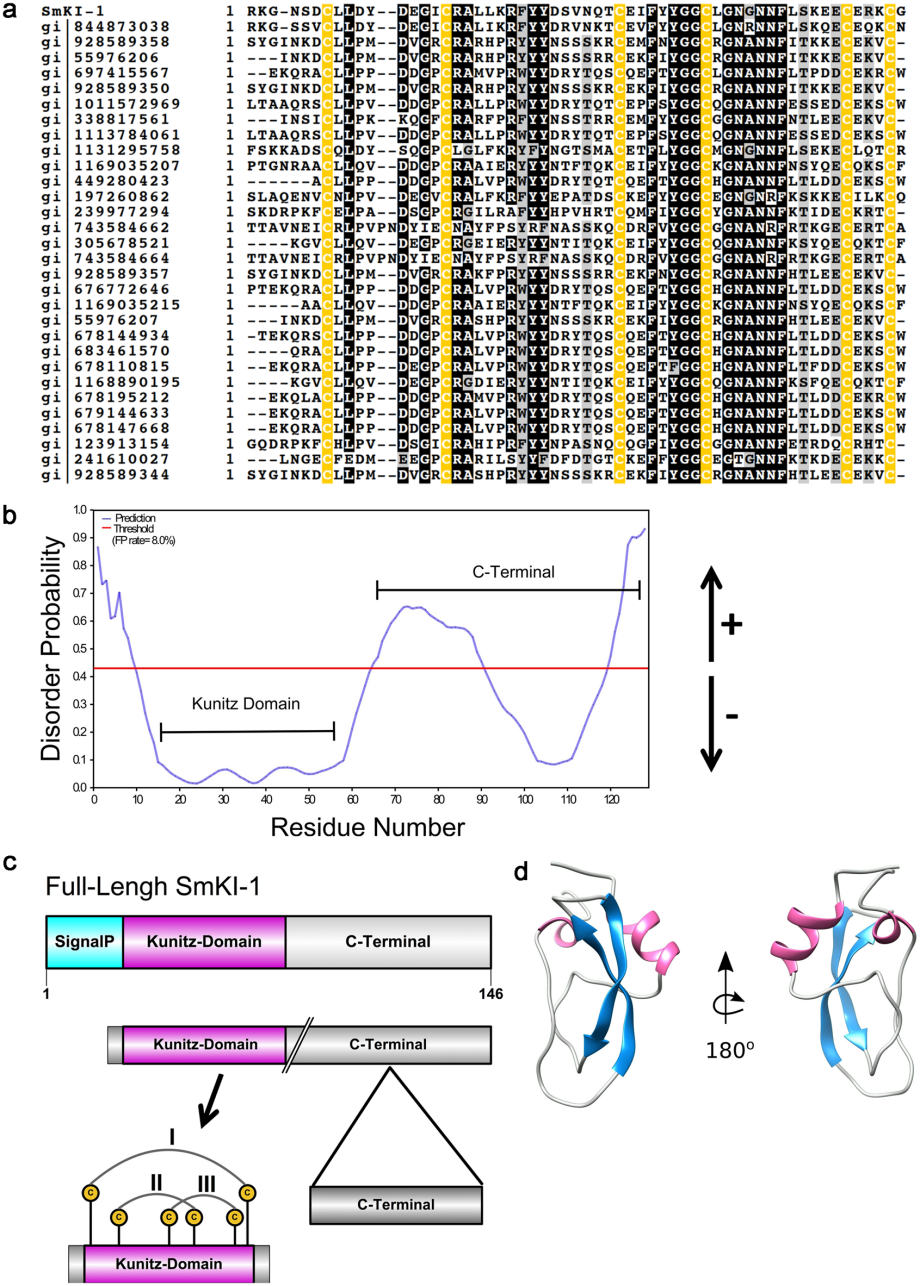
*Sm*KI-1 Kunitz type domain sequence and structure. (**a**) Multiple Sequence Alignment between the SmKI-1 protein from *Schistosoma mansoni*, and its homologous proteins performed using Clustal Omega, refined using BoxShade and then manually. The residues that are similar are shaded in gray, identical in dark-black and in yellow absolutely conserved cysteine residues. (**b**) Disordered Probability Prediction showing the structured Kunitz domain and unstructured C-terminal region. Analysis performed using COILS algorithms available at the Expasy website. (**c**) Schematic representation and linear view of the domains of the full-length SmKI-1 protein showing the Kunitz domain with the three disulfide bounds arrangements. (**d**) 3D protein structure of the *Sm*KI-1 Kunitz domain modeled using MODELLER v9.17.

Initially, we performed bioinformatic analysis to evaluate the *S*. *mansoni Sm*KI-1 (Smp_147730) amino acid sequence in order to determine the conservation of its Kunitz domain (KI) compared to other organisms. The sequence alignment using BLAST homology search for the coded protein *Sm*KI-1 compared to other functionally known Kunitz inhibitor proteins from various species showed the conservation of the P1 site typical of trypsin inhibitor (Arg^18^) and the six cysteine residues (represented in yellow on Fig 1A and 1C). The Kunitz domain of *Sm*KI-1 protein was found to be a well-conserved segment with other Kunitz protease inhibitors such as Actitoxin from *Anemonia viridis* (gi|928589358), Tissue Factor pathway 2 from *Tinamus guttatus* (gi|697415567) and the AMBO protein from *Bos indicus* (gi|1131295758) with 58%, 45%, and 55% of identity, respectively. These data together with PSI-BLAST analysis was used as an input for protein modeling. Complementary *in silico* analysis of *Sm*KI-1 amino acid sequence revealed besides the presence of the conserved Kunitz inhibitor domain (N-terminal), also an unstructured C-terminal region (Fig 1B). Also, BLASTp analysis of the predicted C-terminal amino acid sequence revealed no similarity with other proteins deposited in the databank. Furthermore, we predicted the occupancy of *N*-glycosylation and *O*-glycosylation sites by using bioinformatics programs. Our sequence based predictions revealed only one putative *N*-glycosylation site for *Sm*KI-1 protein at Asn^30^, which agrees with other functionally characterized Kunitz inhibitors with high similarity to *Sm*KI-1 that have this single point modification and previous results described by Ranasinghe and colleagues (S1A Fig) [19]. Glycosylation prediction results show no potential *O*-glycosylation sites in the sequence (all prediction confidence scores were lower than 0.5), and the single *N*-glycosylation site annotated for this protein (Asn^30^) is neither in the primary nor secondary binding loop (S1A Fig).

We modeled *Sm*KI-1 Kunitz domain by comparative homology modeling using the MODELLER v9.17 tool and *iTASSER* software (Fig 1D). The suitable template structure for developing the model was obtained using BLAST by setting PDB as the source database for finding the 3D structure templates. The resulting sequences of at least 48% similarity and identity were selected for comparative homology modeling. PDB ID 4BQD was found to have 65% identity with the query sequence and was chosen as the template for modeling the *Sm*KI’s Kunitz domain. The model was found to be reliable and approximately 91% of the residues fell in the favored region in the Ramachandran plot. Moreover, the canonical segments of the primary binding loop of *Sm*KI-1 (DE^14^GICR^18^ALLK) and the secondary binding loop (F^35^YYGGCLG^43^NG^45^NNFL) adopt a well-conserved conformation, which is similar within other Kunitz inhibitors in complex with trypsin and elastase (Fig 1D). Our modeling also suggests that the untypical Glu^14^ could be important to form the complex and allow the hydrophobic P1 Arg^18^ to enter the active site.

Cloning and heterologous expression of the *Sm*KI-1 and its fragments (Kunitz and C-terminal domains), were performed as described in the material and methods section. Recombinant (r) His-tagged *Sm*KI-1 (r*Sm*KI-1), KI and C-terminal domains were purified from bacterial lysates using nickel affinity chromatography, and then analyzed by SDS-PAGE followed by Coomassie blue staining. A single band of the expected size of r*Sm*KI-1 (~20 kDa), KI (~10 kDa) and C-terminal domains (~15 kDa) indicate the success of the purification protocol (S1B Fig). To further evaluate the specificity of the purification procedure, purified r*Sm*KI-1, KI domain and C-terminal region were analyzed by western blot using an anti-His tag monoclonal antibody (S1C Fig). The western blot analysis confirmed that the purified proteins have the histidine tag and the molecular weight was the expected for r*Sm*KI-1, KI and C-terminal domains. Also, the purity and average molecular mass of the refolded recombinant protein (KI domain) was analyzed by mass spectrometry. Our data showed a single homogeny protein with a [M+H]^+^ = 11,200.0 m/z with theoretical average molecular mass of 11,074.0 Da. The precursor charge state M+2H^+^ was also detected (S1D Fig).

The final product was submitted to circular dichroism (CD) analysis in order to rapid determine the secondary structure and folding properties of this inhibitor. Because the spectra of proteins are so dependent on their conformation, CD can be used to estimate the structure of unkown proteins and monitor their structures. Here, we were able to verify that KI domain was soluble and well-folded and suitable for our assays (S1E Fig). The protein showed a typical negative band at 215 nm and small positive band 195 nm, which is typical for antiparallel β-sheets and other Kunitz-inhibitors [20, 21]. In order to evaluate the cytotoxicity potential of r*Sm*KI-1, we incubated BHK-21 cells with different concentrations of this protein and submitted them to the MTS assay. BHK-21 cells incubated with the amounts of r*Sm*KI-1 used in this study did not show cell death, which was only observed at higher r*Sm*KI-1 concentrations (1000 μg/mL) (S1F Fig). A solution of cetyltrimethylammonium bromide (CTAB), a highly toxic surfactant, was used as a positive control and the BHK-21 cells incubated with medium alone were used as a negative control to calculate the percentage of viable cells. These results supported the safety of r*Sm*KI-1 for the next experiments.

### *Sm*KI-1 and its Kunitz domain but not the C-terminal region inhibit both trypsin and elastase activities

**Fig 2:**
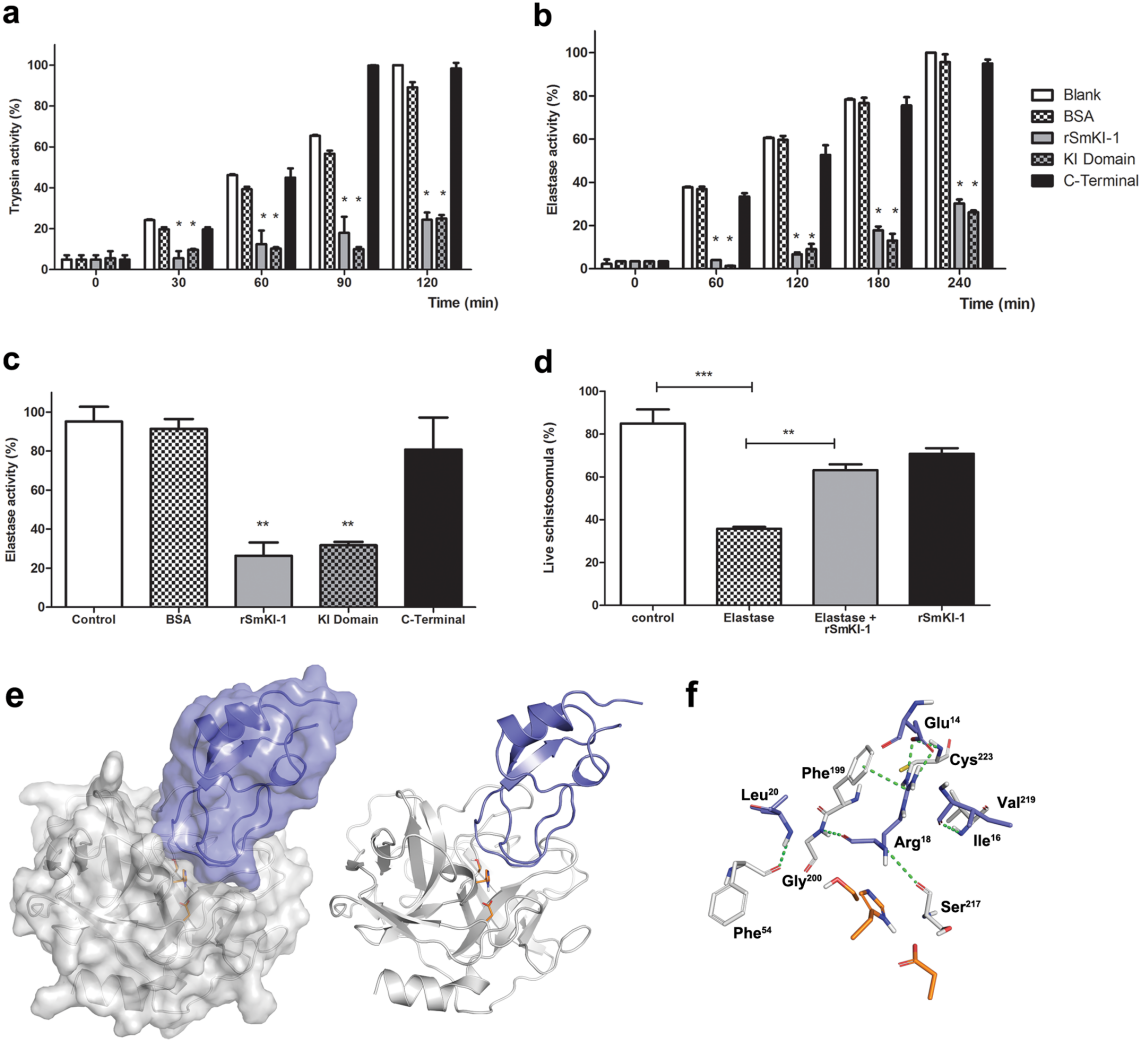
Recombinant *Sm*KI-1 and its Kunitz domain inhibit serine proteases and protect *S*. *mansoni* against neutrophil elastase. Recombinant *Sm*KI-1, its Kunitz or C-terminal domains (100 nM) were tested as inhibitor of serino proteases: (**a**) Bovine Trypsin activity (100nM), (**b**) Human Neutrophil Elastase activity (300nM) and (**c**) Neutrophil-secreted elastase activity. In all experiments, bovine serum albumin (BSA, 300nM) was used as a negative control. Enzyme inhibition was detected over two-hour incubation with r*Sm*KI-1 or its Kunitz domain. Bars indicate each enzyme activity mean ± standard deviation. (**d**) Protective effect of rSmKI-1 (0.15 mg/mL) in cultured schistosomula treated with purified elastase (0.05 mg/mL). Bars represent live parasites ± standard deviation. Data are representative of at least three independent experiments. For (**a**) and (**b**), an asterisk indicate statistically significant differences of r*Sm*KI-1 or Kunitz domain compared to control group p< 0.05. For (**c**) and (**d**), ** asterisks indicate statistically significant differences of r*Sm*KI-1, compared to control group or elastase group p< 0.005. **(e)** Binding mode of *Sm*KI-1 Kunitz domain (purple) to neutrophil elastase (gray) predicted by docking with CLUSPRO 2.0. Residues from elastase catalytic triad (His^70^, Asp^117^ and Ser^202^) are highlighted in orange sticks. (**f**) Detailed analysis of the docking predicted interface reveals residues involved in hydrogen bonds, a salt bridge and a π-stacking interaction (all interactions shown as green dashes). *Sm*KI-1 residues are represented and labeled in purple, NE residues in gray.

Herein, we investigated the *Sm*KI-1 potential to inhibit bovine trypsin and also neutrophil elastase, a well-known human serine protease with important role in inflammation. We performed enzymatic assays *in vitro* using the recombinant (r) full *Sm*KI-1, the Kunitz domain and the C-terminal region of the protein and observed that r*Sm*KI-1 and its Kunitz domain possess inhibitory activity against trypsin (100nM) and neutrophil elastase (300nM) (Fig 2A and 2B). However, the C-terminal domain showed no inhibitory activity. In addition, we observed a reduction in the elastase activity when cultured murine neutrophils were pre-treated with r*Sm*KI-1 or its Kunitz domain and stimulated with N-formylmethionyl-leucyl-phenylalanine (fMLP) (Fig 2C). As observed in *in vitro* assays, the C-terminal domain did not affect the elastase activity. Together, these results strongly suggest that r*Sm*KI-1 anti-elastolytic activity resides on its Kunitz domain and it is independent of the C-terminal region of the molecule.

Previously, neutrophil elastase was found to be toxic to *S*. *mansoni* larvae (schistosomula), since inhibitors prevented elastase-mediated schistosome killing [22]. Then, we decided to test the protective effect of *Sm*KI-1 in cultured schistosomula treated with elastase. The toxic effect of elastase in schistosomula was evident at low concentration such as 0.05 mg/mL. After 24 hours of treatment, r*Sm*KI-1 (0.15 mg/mL) markedly inhibited the capacity of neutrophil elastase to kill schistosomula (Fig 2D). This protective effect observed by r*Sm*KI-1 was probably due to elastase activity inhibition.

To better understand the molecular basis of neutrophil elastase (NE) inhibition by S*m*KI-1 Kunitz domain, molecular docking studies were conducted using the webserver ClusPro 2.0. Docking results suggest a complete blockage of NE active site by *Sm*KI-1, revealing a predicted binding mode very similar to the observed for other elastase inhibitors, in which two *Sm*KI-1 loops (Asp^13^ to Leu^21^ and Phe^36^ to Leu^42^) are in close contact with the enzyme (Fig 2E) [23, 24]. Analysis of the predicted interface with PISA reveals that 14 *Sm*KI-1 residues are involved in the interface, and approximately 20% (761.8 Å^2^) of the inhibitor solvent-accessible area is buried upon complex formation. The interface is also stabilized by five intramolecular hydrogen bonds, all involving residues from the Asp^13^-Leu^21^ loop. Located at the P1 position, *Sm*KI-1 Arg^18^ seems to be a key residue for stabilizing the interaction due to its hydrophobic tail, which can form crucial H-bonds in the S1 pocket. Both its backbone NH and O are involved in hydrogen bonds, respectively to NE Ser^217^ and Gly^200^ (Fig 2F). It has been previously discussed that the P1 position hydrophobic residues are preferred for elastase inhibition, partially due to the difficulty to accommodate bigger residues at S1. In agreement to that, data from the MEROPS database indicates Val, Ile and Ala as the most frequent P1 residues in NE substrates [25]. Also, the Lys13Leu mutation at P1 of a Kunitz inhibitor has been shown to improve affinity to pancreatic elastase [23]. Interestingly, docking results indicate that NE elastase nicely accommodates *Sm*KI-1 Arg^18^ at S1 pocket, since the hydrophobic portion of its side chain occupies the S1 pocket as it would expected for a small hydrophobic residue, while the guanidine group forms an intramolecular salt bridge interaction with *Sm*KI-1 Glu^14^ and a π-stacking interaction to NE Phe^199^. *Sm*KI-1 Glu^14^ is also involved in a hydrogen bond interaction to NE Cys^223^. The remaining predicted hydrogen bonds involve *Sm*KI-1 Ile^16^ and Leu^20^ (bound respectively to NE Val^219^ and Phe^54^). The side chain of *Sm*KI-1 Leu^20^, a highly conserved residue among Kunitz type inhibitors, also fits well in the S2’ elastase pocket (Fig 2F). Altogether, the docking results indicate very good shape and chemical complementarities between *Sm*KI-1 and neutrophil elastase, in agreement with the potent *in vitro* inhibition observed in this study.

### *Sm*KI-1 gene expression is important for *S*. *mansoni* survival

**Fig 3:**
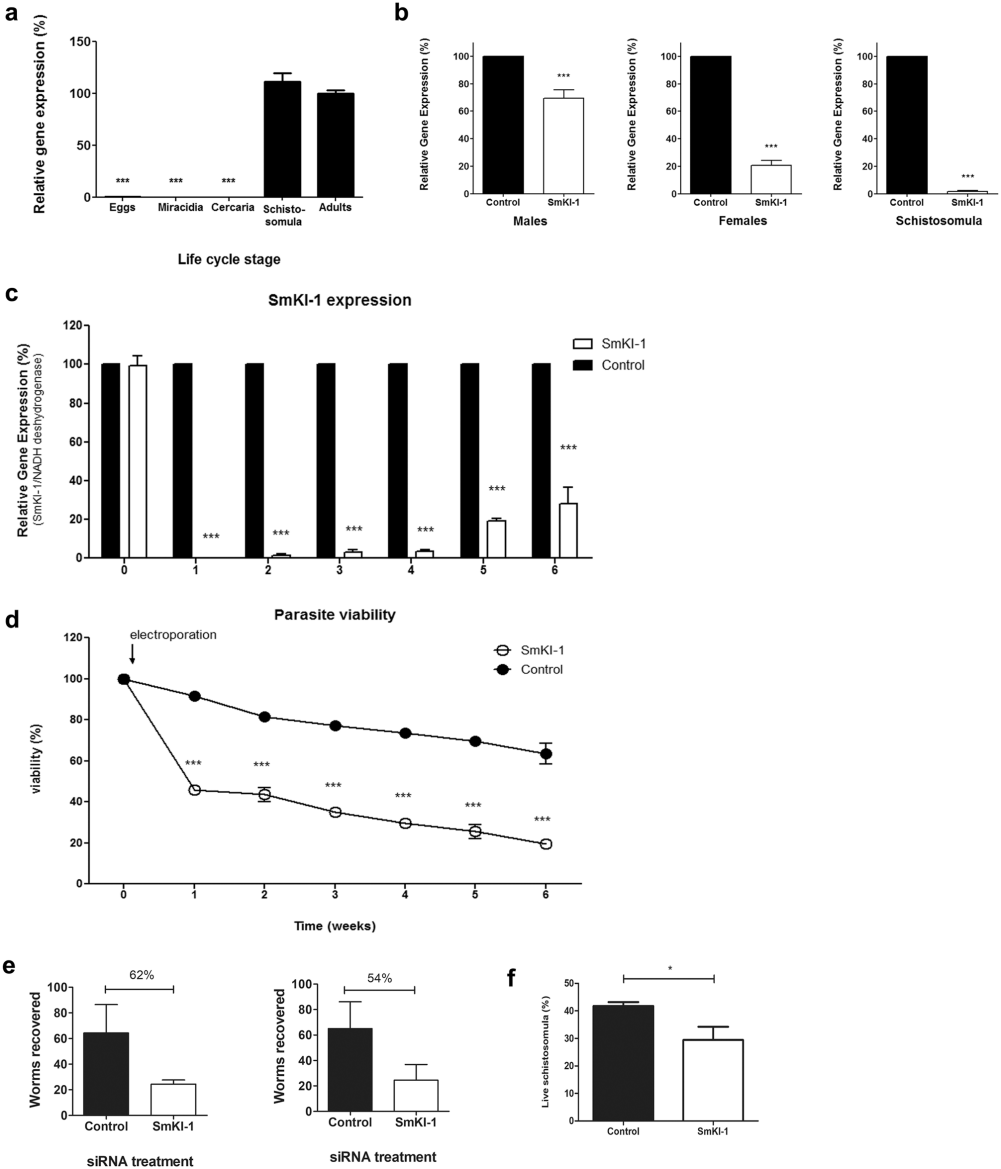
*Sm*KI-1 is important for parasite development. (**a**) Quantitative RT-PCR data showing relative expression level (mean ± SD) of *SmKI-1* at different stages in the *S*. *mansoni* life cycle: eggs, miracidia, cercariae, schistosomula (7-day cultured larvae) and adult worms (male and female—set at 100%). (**b**) Mean level of *SmKI-1* gene expression (±SD, n = 3) in cultured adult schistosome males (left), females (center) or schistosomula (right) at 72 hours after treatment with control siRNA (black bars, set at 100%) or siRNA targeting *Sm*KI-1 (white bars), as determined by qPCR. (**c**) *SmKI-1* gene expression and **(d)** schistosome viability in cultured schistosomula at different time points after treatment with *SmKI-1* siRNAs. White circles/bars represent the relative expression of the group treated with *SmKI-1* siRNAs compared to the group treated with a control siRNA (set as 100%, black circles/bars) at the same time points. Results are representative of two independent experiments. Significant differences between male adult worms and other life stages is denoted by ***, p <0.001. (**e**) Schistosomula were treated with *Sm*KI-1 siRNA and used to infect mice. Worm burden recovery (mean±SD) from two independent experiments are shown. (**f**) Viability of schistosomula treated with *Sm*KI-1 siRNA or control siRNA cultured with mouse neutrophils (mean±SD). Significant differences between groups is denoted by an asterisk, p <0.05. Results are representative of two independent experiments.

In order to investigate the role of *Sm*KI-1 in the parasite, we measured the relative expression of *Sm*KI-1 mRNA in different life cycle stages (e.g.: eggs, miracidia, cercariae, schistosomula and adult parasites) of *S*. *mansoni* by quantitative real-time PCR (qRT-PCR) and the results are shown in Fig 3A. The *SmKI-1* gene exhibited high relative expression in the intravascular life stages, with highest mRNA levels observed in larval and adult parasites. *SmKI-1* transcripts were not detected in eggs, miracidia and cercariae stages. Since *Sm*KI-1 is highly expressed in intravascular stages, we designed specific siRNA in order to knockdown the gene and observed the effects of the lack of *Sm*KI-1 expression in the parasite. In the larval stage, obtained through cercariae mechanical transformation, the siRNA treatment resulted in no detectable expression of *SmKI-1*. However, in adults recovered from infected mice electroporation with *SmKI-1* siRNA resulted in a less efficient suppression: 78% in females and 33% in males, as measured by qPCR (Fig 3B).

*Sm*KI-1-suppressed schistosomula were kept in Braschi medium for six weeks and once a week, gene expression was monitored and the size and viability of suppressed parasites were also evaluated. Suppression rates remained at over 90% up to at least four weeks post *SmKI-1* siRNA treatment. After 6 weeks, the suppressive effect waned although *SmKI-1* gene expression was still 70% lower in suppressed parasites compared to controls (Fig 3C). *Sm*KI-1 suppressed parasites exhibited a 60% reduction in viability when compared to control ones (Fig 3D) and they were also smaller in size (S2A Fig) indicating impaired development under low levels of *SmKI-1* mRNA (S2B Fig).

To investigate whether *SmKI-1* RNAi-mediated gene silencing affected parasite viability *in vivo*, we infected groups of 8 mice with *Sm*KI-1-suppressed schistosomula or control parasites. After six weeks, the adult worms were recovered through perfusion and counted. There was a significant reduction in worm burden in the *SmKI-1*-suppressed parasites compared to the control group, 62% reduction in the first trial and 54% reduction in the second one (Fig 3E). The worm burden reduction also resulted in reduction in eggs found in infected mouse livers (S2C Fig). Since *SmKI-1* gene suppression in larval stage of *S*. *mansoni* robustly impact schistosomes development *in vitro* and *in vivo*, we conclude that expression of this protein is essential for parasite survival.

In order to further investigate the relationship between *Sm*KI-1 and neutrophils, we isolated neutrophils from mice and incubated them *in vitro* with 1-day old schistosomula after treatment with either *Sm*KI-1 siRNA or control siRNA. Following 24 hours of incubation, neutrophils induced schistosomula killing that was significantly higher in *Sm*KI-1-suppressed parasites when compared to control group (Fig 3F). This result suggests a potential role for *Sm*KI-1 to interact with neutrophils to inhibit parasite killing *in vitro*. As a control for viability, both groups of electroporated parasites were also analyzed in the absence of neutrophils and demonstrate no difference in schistosomula survival after the same time in culture (S2D Fig).

Since it is known that neutrophils adhere to the larval schistosomes’ surface and participate in the removal of glycocalyx by phagocytosis *in vivo*[26] and our studies indicates that *Sm*KI-1 seems to impair elastase secretion by neutrophils, we depleted neutrophils *in vivo* and infected mice with *Sm*KI-1-suppressed schistosomula or control parasites. When worms recovered from animals infected with *Sm*KI-1-suppressed schistosomula were compared to parasites treated with control siRNA in neutrophils depleted mice, we still observed a worm burden reduction of 16% suggesting that *Sm*KI-1 plays an important role in parasite development independent of its ability to inhibit host neutrophils *in vivo* (S3 Fig).

### *Sm*KI-1 reduces neutrophil migration and liver damage caused by acetaminophen (APAP)

**Fig 4:**
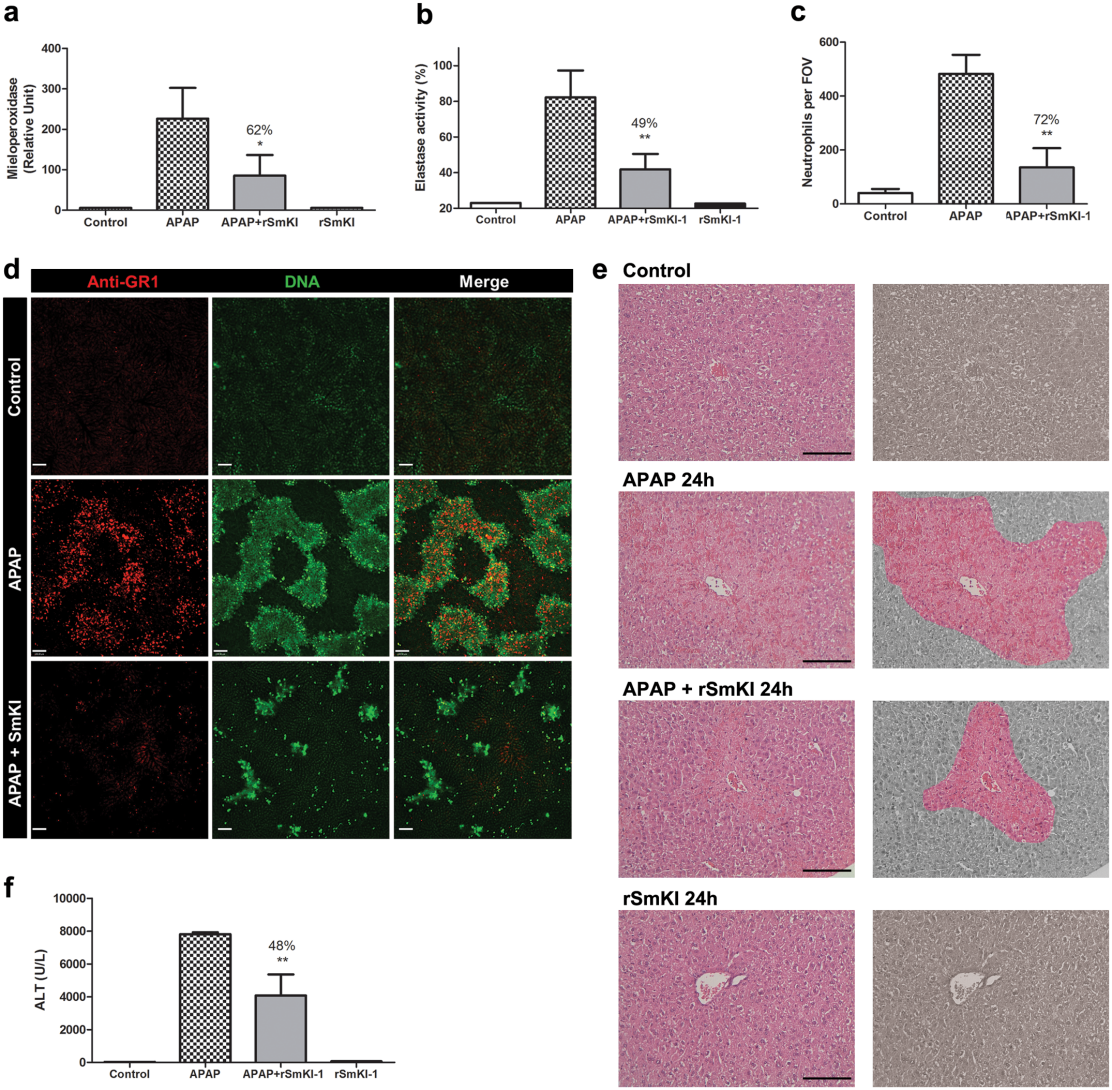
*Sm*KI-1 reduces hepatic APAP-induced injury. **(a)** MPO and **(b)** Elastase activities were measured in r*Sm*KI-1 treated mice during liver APAP-induced hepatotoxicity. Treated-mice received *Sm*KI-1 (10 mg/kg) or PBS vehicle i.v. 15 min prior APAP administration (600 mg/kg). **(c)** Number of neutrophils per field of view (FOV) in the liver of r*Sm*KI-1 treated animals. **(d)** Liver confocal intravital microscopy showing neutrophil (anti-GR1 PE in red) migration into necrotic sites (Sytox green staining) following 24 hours of APAP challenge. Scale bar = 100 μm. **(e)** Left panels represent histology of hematoxylin and eosin-stained liver sections, scale bar = 300μm. In right panels, liver damage is highlighted. **(f)** serum ALT levels confirmed severe liver damage in APAP group and reduction of liver necrosis in mice treated with APAP+*Sm*KI-1. Results are the mean ± SEM of n = 6 per group. An asterisk indicate statistically significant differences of r*Sm*KI-1 compared to APAP group (p< 0.05) or ** p< 0.005.

To further investigate the ability of *Sm*KI-1 to interfere with neutrophil migration and function, we used the acetaminophen (APAP) model, a hepatotoxic compound that caused cell death and massive DNA deposition in liver, inducing acute inflammation associated with neutrophil recruitment [27]. TLR4 KO mice were used in all *in vivo* experiments related to the murine models of inflammatory diseases to minimize the cellular response related to the LPS contamination present in *Sm*KI-1. Mice were divided in two groups, one treated with r*Sm*KI-1 intravenously (APAP+ *Sm*KI-1 group, 10mg/Kg), and another group treated with vehicle (PBS, APAP group). Immediately following *Sm*KI-1 or vehicle injection, mice were challenged with APAP by gavage and samples collected after 24 hrs. Neutrophil migration to mouse livers in response to APAP challenge was first indirectly evaluated through mieloperoxidase (MPO) activity. Mice challenged with APAP had a significantly increased MPO activity when compared to PBS-control group. Previous treatment with r*Sm*KI-1 significantly reduced (62%) MPO activity induced by APAP treatment (Fig 4A). We also analyzed elastase activity and observed an approximately 49% decrease in enzymatic activity in APAP+r*Sm*KI-1 group in comparison to APAP group alone (Fig 4B). To directly investigate the effect of *Sm*KI-1 in neutrophil migration to the liver, we imaged hepatic microenvironment under confocal intravital microscopy. APAP-treated group had extensive areas of DNA accumulation within liver sinusoids and parenchyma and a higher number of infiltrating neutrophils (Fig 4C and 4D). In contrast, APAP+*Sm*KI-1 group had robustly reduced DNA deposition and an approximately 72% lower hepatic neutrophil infiltration (Fig 4C and 4D). Under histopathology analysis, APAP+*Sm*KI-1 treated mice presented milder histological signals of hepatotoxicity (Fig 4E). Conversely, APAP treated animals presented overt liver necrosis and hepatocyte degeneration (Fig 4E). Finally, we investigated serum alanine aminotransferase levels (ALT), a gold standard methodology to access hepatic injury. Serum ALT levels were significantly higher in APAP-challenged mice in comparison to the PBS-control group (Fig 4F). However, APAP+*Sm*KI-1 treated mice had lower ALT levels (48%), indicating decreased hepatic damage (Fig 4F). Taking together, these data shown that r*Sm*KI-1 administration led to reduced liver neutrophil migration and inflammation, culminating in a markedly protection against liver injury triggered by APAP challenge.

### *Sm*KI-1 diminishes inflammatory response in MSU-induced gout

**Fig 5:**
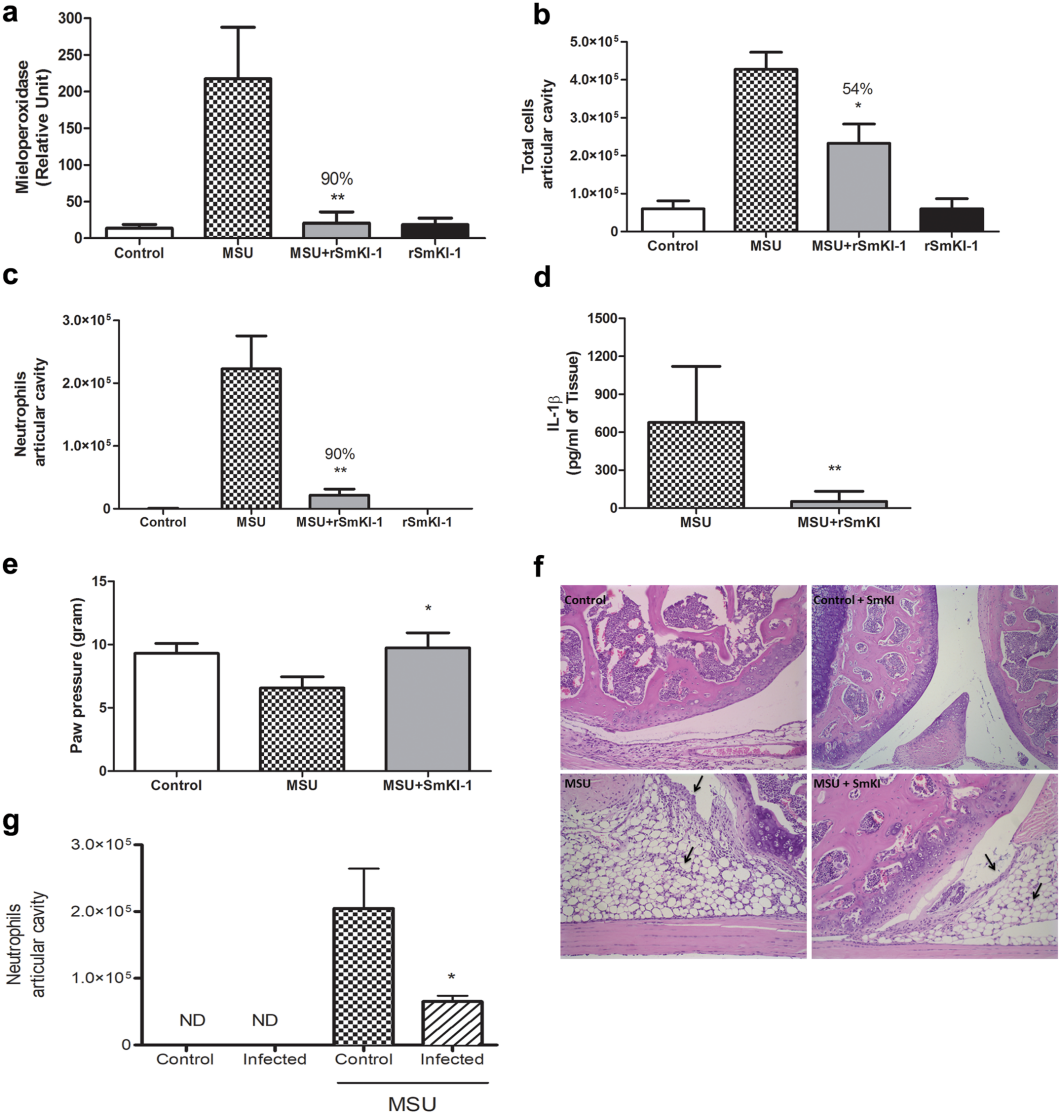
*Sm*KI-1 treatment decreased inflammation after MSU-induced gout. Mice were treated with r*Sm*KI-1 (10 mg/kg) or PBS vehicle i.v. 15 min prior MSU injection. Then, animals were challenged with intra-articular knee injection of MSU (100μg/cavity). Mice were grouped as MSU control, PBS control, MSU+*Sm*KI-1-treatment and PBS+*Sm*KI-1-treatment. Tissue inflammation was evaluated by **(a)** relative numbers of neutrophil in periarticular tissue determined by MPO assay, **(b)** total cells and **(c)** neutrophil recruitment in the synovial cavity, **(d)** IL-1β production measured by ELISA in the periarticular knee tissue and **(e)** joint dysfunction as noted by the increase nociceptive response of mice to mechanical stimulation using an electronic paw pressure meter test 15 hrs after MSU or PBS (control vehicle) injection. **(f)** Representative photographs of hematoxylin and eosin-stained sections of knee joints of mice after 15 hrs of injection with vehicle or MSU crystals (100μg/joint). Leukocyte infiltration and hyperplasia of the synovial membrane are indicated by black arrows. (**g**) Neutrophil recruitment in the synovial cavity of mice infected with *S*. *mansoni*. Mice were grouped as PBS control, PBS infected, MSU control and MSU infected. ND = not detected. Results are the mean ± SEM of n = 6 per group. Asterisks indicate statistically significant differences of r*Sm*KI-1 compared to MSU-vehicle group *p< 0.05 or ** p< 0.005. An asterisk also indicates statistically significant differences of *S*. *mansoni* infection versus control mice that received MSU, p<0.05.

Since *Sm*KI-1 seems to affect neutrophil activity, we then tested its potential to control inflammation, in an experimental model of gout arthritis, in which neutrophils are one of the pivotal inflammatory cells that contribute to disease pathogenesis. Mice were divided in two treated groups, one group was treated with r*Sm*KI-1 (10mg/Kg) intravenously, while the other group received PBS as vehicle. Then, we injected highly pure monosodium urate (MSU) crystals directly into C57BL/6 mice right knee joints and PBS was administered to the left knee as control. Injection of MSU in the knee joint of mice induced significant accumulation of neutrophils in periarticular tissue, as assessed by MPO assay. In contrast, *Sm*KI-1 administration decreased MPO (90%) in the knee joints (Fig 5A). Furthermore, the number of total cell were significantly lower in the articular cavity of *Sm*KI-1-treated mice (54%) as compared to non-treated MSU-challenged mice (Fig 5B). This total cell reduction in articular cavity was accompanied by decrease (90%) in neutrophil infiltration (Fig 5C). Next, we also examined the production of the cytokine IL-β in the knee periarticular tissue and observed markedly increased levels of this cytokine after MSU injection. Conversely, treatment with r*Sm*KI-1 significantly decreased levels of IL-1β (Fig 5D). Additionally, 16 hrs after MSU injection, MSU crystals induced mechanical hypernociception (as observed by the decreased paw withdrawal threshold) due to joint inflammation specially by the neutrophil recruitment, given that neutrophil’s accumulation in the articular knee cavity have been extensively associated with increase hypernocipetion in mice with arthritis [28–30]. However, MSU+*Sm*KI-1-treated mice showed reduced hypernociception (Fig 5E) that was also associated with reduction of neutrophils in the articular knee cavity (Fig 5C). The dorsiflexion-elicited withdrawal threshold was expressed in grams (g) and used to infer behavioral responses associated with experimental pain (hypernociception). The higher withdrawal threshold means lower hypernociception, since mice with reduce pain support more local pressure. Additionally, knee tissue from r*Sm*KI-1 treated mice showed reduction in joint destruction characterized by diminished leukocyte infiltration and hyperplasia from the synovial membrane after MSU injection (Fig 5F).

Finally, we decided to address whether schistosomes could modulate MSU-induced arthritis. After 56 days of *S*. *mansoni* infection, mice were challenged with MSU to induce gout. Interestingly, a significant reduction of neutrophils in the articular knee cavity was observed (Fig 5G) suggesting that the parasite infection is able to modulate the gout inflammatory response, similarly to what was observed when we used r*Sm*KI-1.

### *Sm*KI-1 reduces neutrophil migration in pleural cavity induced by carrageenan

**Fig 6:**
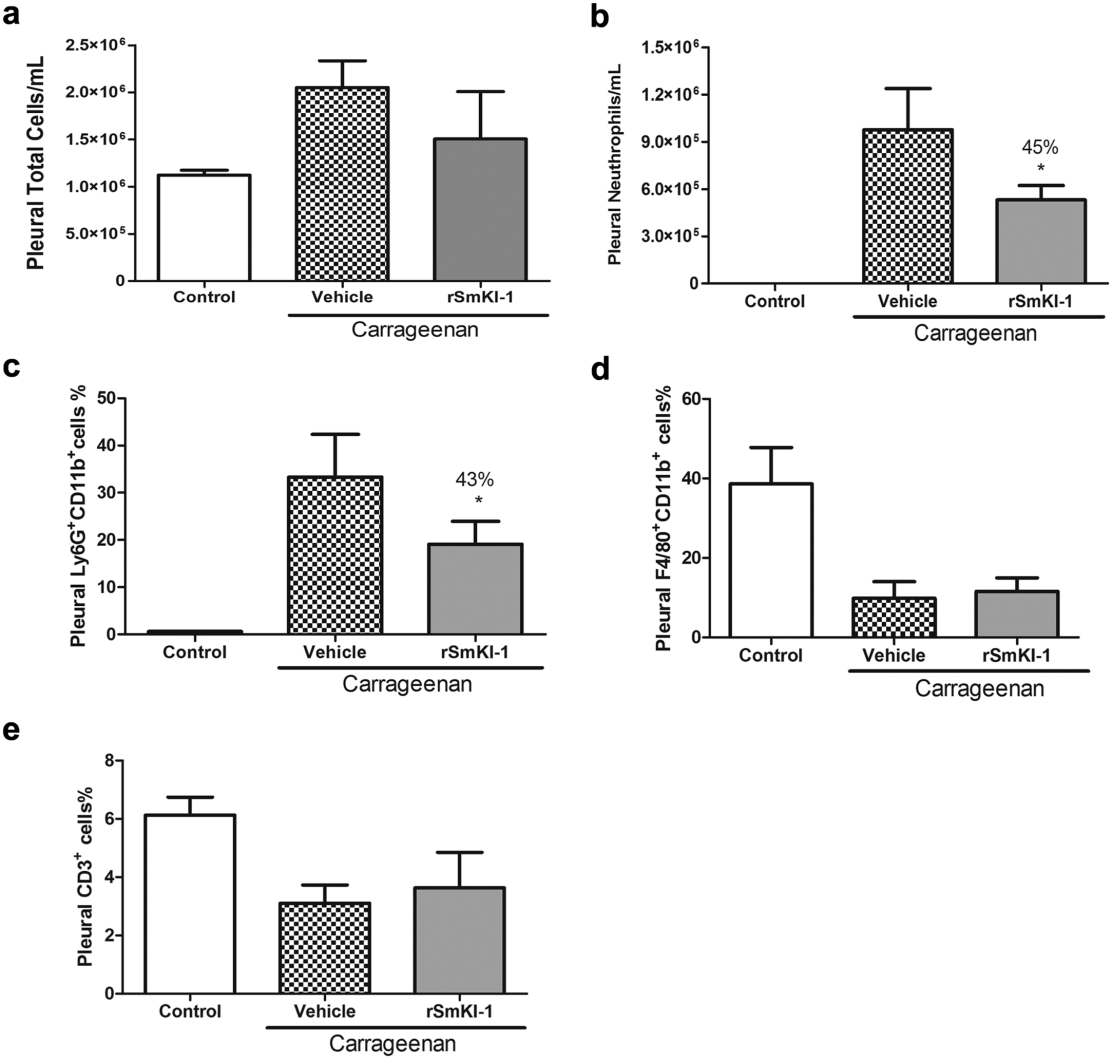
*Sm*KI-1 treatment reduces neutrophil migration into pleural cavity in response to carrageenan injection. After carrageenan injection (2mg/mL) into pleural cavity, animals received an intravenous dose of *Sm*KI-1 (10 mg/kg) or PBS (vehicle). Four hours later, we recovered cells by washing pleural cavity with PBS. Counting of (**a**) total cells and (**b**) neutrophils were performed by cytospin preparations. Specific cell populations in pleural fluid were also evaluated by flow cytometry, being the percentage of (**c**) neutrophils (Ly6G^+^CD11b^+^), (**d**) macrophages (F4/80^+^CD11b^+^), and (**e**) T lymphocytes (CD3^+^ cells) calculated from the total cell numbers. Results are expressed as the number of cells per cavity or percentage of cell subpopulations (mean ± SD) for each treated group (5–6 mice each). An Asterisk indicates statistically significant differences of carrageenan+*Sm*KI-1 compared to carrageenan vehicle group (p< 0.05).

To confirm that r*Sm*KI-1 inhibits neutrophil migration, we used the carrageenan-induced pleurisy model that induces an inflammatory response promoted by neutrophil adhesion and migration through the vascular endothelial cells [31]. The fluid leakage from the mouse pleural cavities was analyzed by cytospin and flow cytometry to determine the presence of cells after carrageenan-induced inflammation. First, animals were injected with carrageenan (2mg/mL) and then treated with r*Sm*KI-1 (10 mg/kg) or vehicle. Then, we evaluated the migrating cells to the pleural cavity between two mouse groups, the carrageenan+*Sm*KI-1 treated group and the carrageenan alone group. There was no significant difference in total number of cells infiltrating into the pleural cavity after intrapleural injection of carrageenan in both experimental groups (Fig 6A). However, we observed that r*Sm*KI-1 treatment reduced around 45% of neutrophil recruitment to the pleural cavity of mice after carrageenan administration as measured by cytospin (Fig 6B). Similar findings were obtained (43% reduction) when we evaluated the percentage of neutrophil population (Ly6G^+^CD11b^+^) by flow cytometry (Fig 6C). Additionally, we demonstrated that *Sm*KI-1 treatment after carrageenan intrapleural injection did not interfere with the percentage of carrageenan-induced macrophages (F4/80^+^CD11b^+^) (Fig 6D) and T lymphocytes (CD3^+^ cells) (Fig 6E) recruited into the pleural cavity compared to the control vehicle.

### *Sm*KI-1 does not affect lymphocyte and macrophage migration in peritoneal cavity induced by thioglycollate

To confirm that r*Sm*KI-1 does not alter a model of neutrophil-independent inflammation, we used the thioglycollate-induced nonspecific cell migration. As demonstrated by Ray and Dittel [32], the peritoneal cavity harbors a number of immune cells, especially macrophages and lymphocytes. First, animals were injected with thioglycollate (3mL of 3% solution) and then treated with r*Sm*KI-1 (10 mg/kg) or vehicle. Then, we evaluated the cells present in the peritoneal cavity by cytospin between two mouse groups, the thioglycollate+*Sm*KI-1 treated animals or the thioglycollate alone administered group. There was no significant difference in total number of cells infiltrating in both experimental groups (S4A Fig). Similar findings were obtained when we evaluated the lymphocyte (S4B Fig) and macrophage (S4C Fig) populations, suggesting that the administration of r*Sm*KI-1 does not promote a broadly immunosuppressive effect, since it does not affect the migration of lymphocytes and macrophages in both thioglycollate and carrageenan-induced inflammation models.

## Discussion

Serine proteases are involved in different physiological aspects such as blood coagulation, apoptosis, signal transduction, wound healing, proteolysis cascade action and inflammatory responses [33]. Particularly, elastase is a serine protease produce by activated neutrophils, and secreted during inflammation as well as in the initiation of blood coagulation [17, 34]. Furthermore, neutrophil elastase is an important host defense molecule required for protection against pathogenic bacteria and fungi [35]. Regarding schistosomes, a previous study demonstrated that neutrophil elastase is toxic to the larval stage of this parasite [22]; however, the mechanisms by which elastase kills a multicellular organism such as the schistosomes are not known. *S*. *mansoni* infect humans and use proteases to invade host tissues and for nutrition and development. Additionally, adult schistosomes live in the blood vessels of the mammalian hosts and survive in this hostile environment without triggering immune attack by immune cells, such as neutrophils or inducing complement activation [36]. Since serine proteases are the most abundant protease family represented in the human genome, we can hypothesize that helminth parasites, such as *S*. *mansoni*, have evolved to survive in their human hosts by producing parasite-derived protease inhibitors that specifically target human proteases.

Herein, we demonstrated that *Sm*KI-1 acts as a trypsin and neutrophil elastase inhibitor *in vitro*. Previously, Ranasinghe et al. was the first to describe *Sm*KI-1 and they reported the potential anti-coagulant and anti-inflammatory properties of this molecule [19]. We then took further and also evaluated the inhibitory activity of its Kunitz domain and C-terminal region of the protein as individual molecules. In this study, we demonstrated that the protease inhibitor function of *Sm*KI-1 is due to its Kunitz domain, but not the C-terminal region of the molecule. Elastase secreted by cultured neutrophils was also inhibited by *Sm*KI-1 and its Kunitz domain, demonstrating that this molecule also robustly interferes with the enzyme activity of elastase released by murine neutrophils. Elastase is a key enzyme in the mammalian host immune system, being important in defense against different pathogens. Belaaouaj and colleagues [37] showed that cleavage of outer membrane protein A by neutrophil elastase resulted in *Escherichia coli* cell death. In this study, we also demonstrated that *Sm*KI-1 reduces schistosomula killing by elastase. Therefore, the ability of *Sm*KI-1 to interact with elastase’s catalytic site as demonstrated by our docking experiments and to inhibit enzyme activity could be a potential immune evasion mechanism developed by the parasite through evolution.

Furthermore, we demonstrated that *Sm*KI-1, the first Kunitz-type serine protease inhibitor described in *S*. *mansoni*, has the highest relative expression in the intravascular life stages, larval and adult parasites. Higher *SmKI-1* mRNA expression in adult parasites but not in larval stage was previously reported [19]. We hypothesized here that *Sm*KI-1 is an important molecule involved in parasite development. To test this hypothesis, *Sm*KI-1 expression was suppressed using siRNA in *S*. *mansoni* adult males, females and schistosomula, being the most efficient suppression observed in the larval stage. The *Sm*KI-1-suppressed schistosomula presented lower viability compared to control parasites when kept in cultured. Additionally, when *Sm*KI-1-suppressed parasites were inoculated to mice 54–62% less helminths were recovered from infected animals. Therefore, *Sm*KI-1 siRNA suppression demonstrated a robust impact in parasite survival inside the mammalian host. One explanation for this outcome may be the observation that *Sm*KI-1 is released in the excretory-secretory products of the *S*. *mansoni* and this molecule is probably involved in providing protection to the parasite *in vivo*. Helminth excretory-secretory products are predominantly comprised of proteases, protease inhibitors, venom allergen homologues, glycolytic enzymes and lectins [38]. These products which act in the host/parasite interface may be important in immune response and immune modulation. *Fasciola hepatica* Kunitz type molecule (Fh-KTM) present in parasite total extract was responsible for suppressing pro-inflammatory cytokine production in LPS-activated dendritic cells (DC) that impaired their ability to induce inflammatory responses [39].

The inhibition of key inflammatory enzymes, such as elastase, in the mammalian host is an important immune modulatory mechanism used by pathogens. In this study, we demonstrated the protease inhibitory activity of *Sm*KI-1. Therefore, we hypothesized this protein is able to modulate inflammation, interfering with neutrophil activity, especially migration. The role of neutrophils in the pathophysiology of inflammatory diseases is well established, with many lines of evidence supporting a direct correlation between the presence of neutrophils in inflamed tissue and the progression of inflammatory diseases [40, 41]. Therefore, we tested the *Sm*KI-1 anti-inflammatory potential in three different disease models: APAP-induced liver injury, MSU-induced gout and carrageenan-induced pleural inflammation.

Drug-induced liver injury is one of the major causes of acute liver failure with yet few therapeutic options. In clinical treatment, acetaminophen (APAP) is frequently used as antipyretic and analgesic, being a safe drug when applied in the therapeutic range (<4g/day). However, unsupervised and long-term abuse of APAP has contributed to increased rates of liver injury and acute liver failure worldwide [42, 43]. In mice, liver injury is induced by a single high dose of APAP [44]. Here, we induced liver damage in order to evaluate the possible effects of *Sm*KI-1 in neutrophil recruitment and activation since these cells play a major role in acute injury development. As expected, the administration of APAP caused a striking congestion of mouse liver cells, associated with necrosis and accumulation of erythrocytes. We used confocal intravital microscopy to follow directional migration of neutrophils to liver due to APAP overdose. Treatment with *Sm*KI-1 rescued APAP-mediated liver damage, with a significant reduction in both neutrophil recruitment and elastase activity.

Gout arthritis is an inflammatory disease caused by the deposition of MSU crystal in the articular junctions [45]. MSU crystal promotes the production of chemokines and cytokines and expression of cell adhesion molecules, which induce the migration of inflammatory cells into sites of tissue injury [46]. *Sm*KI-1 was used to treat mice after MSU-induced gout. This protein reduced neutrophil accumulation, hypernociception, and overall pathological score. The diminished joint damage was associated with decreased local production of the pro-inflammatory cytokine, IL-1β, one of the cytokines responsible for the initiation and perpetuation of the inflammatory response which promotes neutrophil influx to the sites of tissue injury [47]. These findings demonstrate, for the first time, the potent anti-inflammatory activity of *Sm*KI-1 in experimental gout arthritis. Other studies have proved that some helminthic infections strongly influence the immune system and enable protective pathways in immune disorders, like arthritic disease [48, 49]. Parasite infection has shown to decrease the severity of collagen induced arthritis in mice through local and systemic suppression of pro-inflammatory mediators, suggesting their substantial benefit as therapeutic agents against rheumatoid arthritis [48]. Fortunately, the study of parasite recombinant proteins demonstrate that regulation of immune response is not strictly dependent on parasite infection, but is associated with pathogen-derived antigens [50]. The r*Sm*KI-1 is one example of a *S*. *mansoni* molecule that possesses a robust anti-inflammatory activity against gout arthritis.

Lastly, we used carrageenan, a strong inflammatory agent that induces the production of leukotriene (LT) B4 at the site of inflammation [51]. Using the carrageenan-induced pleural inflammation model, we demonstrated the ability of *Sm*KI-1 to inhibit early events that trigger neutrophil recruitment in the pleural cavities of mice in response to inflammatory stimuli. Although our results indicate the effect of *Sm*KI-1 on neutrophil recruitment and function, we still need to fully understand the mechanisms involved in its anti-inflammatory activities. Evidences point out to impairment of neutrophil recruitment due to elastase inhibition, but more studies are required to confirm this hypothesis. Our findings have a strong impact on drug development that inhibit neutrophil migration and it may represent an important new therapeutic strategy for treatment of inflammatory diseases [52].

In summary, the data presented here demonstrated that *Sm*KI-1 is a key protein in *S*. *mansoni* survival and maintenance inside their mammalian hosts. This protein possesses the ability to inhibit neutrophil elastase activity and also the migration of these cells in different models of inflammatory diseases, leading to an interest in its potential as a drug for disease treatment. Additionally, we demonstrated that *Sm*KI-1 function is related to the Kunitz domain present on its protein structure. Based on our understanding of the pathogenesis of inflammation, multiple pharmacological interventions have been developed in the past several decades. Although effective, none of the anti-inflammatory agents have had a lasting effect on disease control. Therefore, the results obtained here encourage us to support further studies using r*Sm*KI-1 as a promising therapeutic molecule against inflammatory diseases.

## Materials and methods

### Ethics statement

All experiments involving animals were conducted in accordance with the Brazilian Federal Law number 11.794, which regulates the scientific use of animals in Brazil, the Institutional Animal Care and Use Committees (IACUC) guidelines and the Animal Welfare Act and Regulations guidelines established by the American Veterinary Medical Association Panel on Euthanasia. Animals were fed, housed and handled in strict agreement with these recommendations. All protocols were approved by the Committee for Ethics in Animal Experimentation (CETEA) at Universidade Federal de Minas Gerais UFMG under permit 179/2010.

### Mice and parasites

Female C57BL/6 and TLR4 KO mice aged 6–8 weeks were purchased from the Federal University of Minas Gerais (UFMG) animal facility. *S*. *mansoni* (LE strain) cercariae were routinely maintained in *Biomphalaria glabrata* snails at Centro de Pesquisa René Rachou Fiocruz (CPqRR) and prepared by exposing infected snails to light for 2 h to induce shedding of parasites. Cercariae numbers and viability were determined prior to infection using a light microscope prior to infection. Schistosomula were obtained after separation from the tails by centrifugation using a 57% Percoll (Pharmacia, Uppsala, Sweden) solution. Parasite was cultured for at least one day *in vitro* as previously described [53]. Adult worms were obtained by perfusion of the portal hepatic vein from Swiss mice, 6–7 weeks after infection with approximately 125 cercariae [53]. Perfusion was performed using RPMI-1640 media or PBS containing 1.5% sodium citrate. Parasite eggs were recovered from the livers of these mice as previously described [53].

### Chemicals

All reagents were purchased from Sigma-Aldrich, CO (St. Louis, MO, USA) unless otherwise specified.

### In silico analysis and modeling of *Sm*KI-1 Kunitz Inhibitor from *Schistosoma mansoni*

Comparison of the *Sm*KI-1 deduced protein sequence with other Protein database sequences were performed using the BLASTp software [54] from the NCBI databank (http://www.ncbi.nlm.nih.gov). The Conserved Domain Database search (CDD-Search) from the NCBI site was used to compare motif identity and similarity with known conserved domains [55]. Sequence alignments were obtained by using CLUSTAL OMEGA software [56], edited using BIOEDIT and refined using BoxShade. The physical-chemical properties of the deduced protein were determined by the Protein Machine software available at the Expasy website (http://us.expasy.org/tools/). The disorder probability prediction was performed using the COILD algorithms, which calculates the probability that the sequence will adopt a coiled-coil conformation. Due to the nonexistence of experimental 3D-structures of *Sm*KI-1 protein, a hybrid approach to protein 3D-strucutre predictions was chosen to generate an accurate, atomistic structure of the *Sm*KI-1 Kunitz domain. Three major steps were involved in the modeling. First, all the representative Kunitz inhibitors sequences were submitted to ITASSER web-server [57], including specification on a homologous Tissue Factor Pathway Inhibitor (TFPI) (PDB accession code: 4BQD) as an additional template. The model of the highest C-score was then chosen to proceed. Second, the disulfide bridges were minimized using MODELLER v9.17, which is a computer program used for comparative homology modeling of protein structures. MODELLER v9.17 was downloaded from the Andrej Sali laboratory website (https://salilab.org/modeller/). The basic steps involved in homology modeling using MODELLER are the initial template selection using PSI-BLAST, final template selection and alignment of the query sequence with the template structure, building the model based on the final template selected, followed by model evaluation, MD simulation procedures and validation by using Ramachandran Plot.

### Molecular docking of *Sm*KI-1 Kunitz domain to elastase

Prior to docking, the *Sm*KI-1 Kunitz domain was minimized with the software MacroModel (Schrodinger version 2015–3), employing the OPLS_2005 force field, an implicit water solvent model and the Polak-Ribiere Conjugate Gradient (PRCG) minimization method with a maximum of 2500 iterations and convergence based on the energy gradient and a 0.05 kJ/Å-mol threshold. During minimization, disulfide bonds between the three Cys pairs (Cys^7^-Cys^57^, Cys^16^-Cys^40^, Cys^32^-Cys^53^) were kept as constraints. Molecular docking was performed with the webserver ClusPro 2.0, developed for protein-protein docking [58]. The *Sm*KI-1 Kunitz domain model was docked to the human neutrophil elastase structure extracted from a crystallographic complex with an inhibitor from *Staphylococcus aureus* (PDB accession code 4NZL) [24]. The best scoring model, based on the balanced scoring function, was analyzed. Analysis of the predicted protein-protein interface was performed with the “Protein interfaces, surfaces and assemblies” service PISA at the European Bioinformatics Institute (http://www.ebi.ac.uk/pdbe/prot_int/pistart.html) to calculate interactions and interface properties [59]. The complex was also manually analyzed with the software Pymol.

### Construction, expression and purification of *Sm*KI-1, Kunitz and C-terminal domains

The plasmid pJ414 containing the sequence for r*Sm*KI-1 (pJ414::*Sm*KI) was manufactured by DNA 2.0, Inc. USA (https://www.dna20.com) using DNA2.0 optimization algorithms for expression in *Escherichia coli*. Furthermore, the *Sm*KI-1 Kunitz domain (KI domain) and *Sm*KI-1 C-terminal tail (C-terminal) were cloned into a pET28a vector, into the *Nde*I and *Xho*I restriction sites. The polymerase chain reaction (PCR) generated fragments corresponding to KI domain (N-terminal Arg^22^-Thr^82^) or C-terminal (C-terminal Gli^79^-Glu^146^). These plasmids were transformed into *E*. *coli* Rosetta(Merck KGaA, Darmstadt, Germany) competent cells. Cells transformed with expression plasmids of *Sm*KI-1, KI domain and C-terminal were screened on LB agar plates containing ampicillin (50 μg/ml) and chloramphenicol (34 μg/ml). DNA sequencing was performed to confirm the presence and the correct orientation of the open reading frames. The clones were cultured in selective medium on a rotary shaker at 200 rpm at 37 °C to an optical density at 600 nm of approximately 0.5–0.8 and gene expression was induced by using 1mM isopropylthiogalactoside (IPTG). Five hour after induction, the bacterial cells were harvested by centrifugation at 4000 *g* for 20 min. The pellet was resuspended in 50 mL of 10mM Na_2_HPO_4_, 10mM NaH_2_PO_4_, 500mM NaCl and 10mM imidazole. Subsequently, the cells were submitted to three cycles of sonication lasting 30 seconds each and centrifuged at 5400 *g* for 20 minutes. The r*Sm*KI-1, KI and C-terminal domains were recovered as inclusion bodies and solubilized in 50 mL of 8 M urea, 10 mMNa_2_HPO_4_, 10 mM NaH_2_PO_4_, 500 mM NaCl and 40 mM imidazole. The KI domain was recovered from the inclusion bodies solubilized in 6 M GuHCl and centrifuged at 10,000 g for 1 hour. Recovered proteins were loaded into Ni charged Sepharose-resin (Hitrap chelating 5 mL) at room temperature. The column was washed sequentially several times with Tris-Buffer without GuHCl, and finally the refolded protein was eluted with Tris-buffer containing 400 mM imidazole. The protein was further dialysed against buffer without imidazole and purified to homogeneity by reverse phase chromatography using C_8_ column PRP-3 (Hamilton 7.0 mm x 305 mm) with a linear gradient of acetonitrile (5%-95%) containing 0.1% TFA. The resulting samples were freezed-dried under vacuum. The proteins were purified by affinity chromatography on a Ni-Sepharose column (Hitrap chelating 5 mL) under denaturing conditions using an AKTAprimePlus chromatography system (GE Healthcare, São Paulo, Brazil) according to the manufacturer's protocol. Fractions containing these three proteins were determined through SDS/PAGE-20% and they were dialyzed against PBS pH 7.0 for 16 hours at 4°C. The recombinant proteins were quantified using the BCA kit (Pierce, Waltham, USA) and used for all experiments.

### SDS-PAGE and immunoblotting

Purified r*Sm*KI-1, KI and C-terminal domains were analyzed on 20% polyacrilamide SDS-PAGE gel prepared and run as previously described [60]. Proteins were then transferred to a Hybond-P PVDF membrane (GE Healthcare, Pittsburgh, PA, USA) [61]. The membrane was blocked with TBS-T (tris-buffered saline pH 7.5, 0.05% Tween 20) containing 5% dry milk for 16 h at 4 °C. The membrane was then incubated with a mouse monoclonal antibody to the 6XHis-tag (GE Healthcare) (1:2,000) for 1 h at room temperature. After three washes with TBS-T, the membrane was incubated with goat anti-mouse IgG conjugated to horseradish peroxidase (HRP) (1:2,000) for 1 h at room temperature. After three washes, the membranes were developed using Immobilion Western HRP subtract (Millipore Corporation, Billerica, MA, USA) according to the manufacturer’s instructions and visualized in Amersham Imager 600 (GE Healthcare).

### Mass spectrometry analysis and secondary structure determination

The refolded protein (KI domain) purity was analyzed by MALDI-TOF/MS (AutoFlex III, Buker Daltonics, Germany) using close external calibration under linear mode. Approximately 50 nM of soluble protein was dissolved in Milli-Q H_2_O, mixed to a saturated solution of α-cyano-4-hydroxycinnaminic acid, spotted on a MALDI sample plate, and dried at room temperature. All resulting data was analyzed manually using both mMass and Flex Analysis 3.0 (Bruker Daltonics) softwares. Also, the secondary structure of the refolded protein was investigated by circular dichroism using a Jasco-J815 spectropolarimeter (Jasco International Co., Japan). Spectrum was acquired at room temperature from 194 nm to 260 nm as an average of 5 readings using a 0.1 cm path length cell, data pitch 0.2 nm and a response time of 0.5 s. Data scans of buffer solutions were acquired and subtracted from protein data. The spectra was converted to mean residue ellipticity and readings at [Θ]_222_ nm, [Θ]_215_ nm and [Θ]_208_ were used to estimate protein conformation.

### Cytotoxicity analysis of recombinant *Sm*KI-1

To evaluate the cytotoxicity of the recombinant *Sm*KI-1, 5x10^4^ BHK-21 cells were seeded in a 96 well plate in RPMI medium supplemented with 5% fetal bovine serum, 150 U penicillin G sodium and 150 μg streptomycin sulfate and incubated for 16 to 24 h. We removed the medium from the BHK-21 cells and added 100 μL of RPMI medium supplemented with 2% fetal bovine serum, 150 U penicillin G sodium, 150 μg streptomycin sulfate and r*Sm*KI-1 at the final concentration of 1000, 500, 250, 125, 62.5, 31.25 or 15.62 μg/mL. BHK-21 cells were incubated only with medium to calculate the percentage of total viable cells. An aqueous solution of CTAB (100 mM) was used as a positive control of cytotoxicity. The cells were incubated at 37°C with 5% CO_2_. After 24 h of incubation, we evaluated the cell viability using the CellTiter 96 AQ_ueous_ One Solution Non-Radioactive Cell Proliferation Assay (Promega), according to the manufacturer’s instructions.

### *In vitro* serine protease inhibition assays

The kinetics of the inhibition assay were monitored by analyzing variations in optical density of the samples in microplate. For the inhibition reactions of trypsin the enzyme:inhibitor ratio of 1:1 and neutrophil elastase 1:3 were determined. Then, we used the optimal concentration of bovine trypsin (100 nM) (Sigma, St. Louis, MO, USA) that was incubated in 100 mM Tris-HCl (pH 8.0) containing 20 mM CaCl_2_ and 0.05% Triton X-100 with 100nM of rSmKI-1, KI and C-terminal domains at 37°C for two hours. The residual enzyme activity was determined at 405 nm every 30 minutes using the substrate 0.5 mM BApNA (Sigma). Another inhibition test was performed using 100 nM human neutrophil elastase (Innovative Research, Inc., Novi, MI, USA) and 300nM r*Sm*KI-1, at 37°C for two hours in 50 mM Tris-HCl buffer (pH 7.4), and the residual enzyme activity was determined at 405 nm every 30 minutes using 0.5 mM of the substrate S4760 (Sigma). As a negative inhibition control was used 300 nM of Bovine serum albumin (BSA) in all assays.

### Neutrophil isolation

Mice were killed by cervical dislocation, the femurs and tibias from both hind limbs were removed and freed of soft tissue attachments, and the extreme distal tip of each bone extremity was cut off. PBS was forced through the bone by using a 1-ml syringe with a 27-gauge needle. After dispersing cell clumps and removing the debris, the bone marrow cells were centrifuged at 200*g* for 10 min at 4°C, and the pellet was resuspended in PBS. Bone marrow suspended in 2 ml of PBS were laid on top of a three-layer Percoll gradient prepared in 15-ml polystyrene tubing by layering 2 ml each of 1.095, 1.085, and 1.070 g/mL Percoll solutions. After centrifugation at 500*g* for 30 min at 4°C in a swinging bucket rotor, the lowest band (1.085/1.095 g/ml interface) was collected as the neutrophil fraction. Remaining erythrocytes were lysed by hypotonic shock. The resultant pellet was suspended in PBS containing Ca2^+^/Mg2^+^ and 2.5% BSA. In all experiments neutrophil samples were 97% viable as determined by Trypan blue staining.

### Neutrophil elastase inhibition by *Sm*KI-1 and Kunitz domain

Elastase release and inhibition assay of freshly isolated neutrophils (1x10^6^ cells) were stimulated with fMLP (100 nM) for a further 10 minutes before addition of the 40μM of *Sm*KI-1, KI and C-terminal domains at 37°C for one hour. After that, it was added colorimetric substrate S4760 (100 ng/ml, Sigma). Cleavage of substrate was immediately monitored colorimetrically at 405 nm every hour for 16 hours. Each reaction (200 μl) was performed in triplicate in a 96-well plate. Initial reaction rates are reported as the change in absorbance per minute.

### Treatment of schistosomes with elastase

Freshly prepared schistosomulas were washed 3 times and resuspended in RPMI medium. Aliquots containing 150 schistosomula were dispensed into flat-bottom, 96-well microtiter plates and 0.15mg/mL of r*Sm*KI-1 was added for one hour at 37°C and 7.5% CO_2_. Then, 0.05mg/mL of human neutrophil elastase (Innovative Research, Inc., Novi, MI, USA) was added and the volume was brought to 200 μl with RPMI. The microtiter plates were covered and placed in a humidified incubator at 37 °C and 7.5% CO_2_. After 18 hrs, the plates were inspected under an inverted microscope (Olympus Co., Hamburg, Germany). In each well, the percentage of dead schistosomes was scored. Dead schistosomes appear as an opaque and granular appearance, and lacked flame cell activity. This measurement correlated well with a dye-exclusion counting [62].

### Preparation and delivery of *Sm*KI-1-suppressed parasites

Two gene-specific small inhibitory RNAs (siRNAs) were commercially synthesized (Integrated DNA Technologies, Inc.) and used to knockdown *SmKI-1* gene expression. These are si*Sm*KI-1I and si*Sm*KI-1II. The DNA sequence of *Sm*KI-1I is 5’-CTACGACAGTGTAAATCAAACTTGT-3’, spanning coding DNA positions 135–160 and the DNA sequence for *Sm*KI-1II is 5’-GGATGTCTTGGAAATGGAAACAACT-3’, spanning coding DNA positions 178–203, both designed with the help of the online IDT RNAi Design Tool (https://www.idtdna.com/Scitools/Applications/RNAi/RNAi.aspx). The siRNAs were delivered to 1-day or 7-day old schistosomula or adult parasites by electroporation as previously described [63]. The control, irrelevant siRNA is: 59-CT TCCTCTCTTTCTCTCCCTTGTGA-39. To monitor gene expression at various times after siRNA administration, qRT-PCR was performed using custom TaqMan Assays. Schistosomula cultures were monitored along six weeks to compare control and *SmKI-1* siRNA-electroporated parasite size, images were taken using an inverted microscope (EVOS FL Cell Imaging, Life Tecnologies, Carlsbad, CA, USA) and parasite viability in culture was measured by adding 1 mg/ml Hoechst 33258 to the cultures at room temperature. After 10 min dead parasites (Hoechst positive) were counted microscopically, using a 460 nm reading filter.

For *in vivo* analysis, two groups of female C57BL/6 mice (10 animals per group) were included in this study. Each group received one dose of 50 μL containing 500 schistosomula in a quadriceps muscle. One group received 500 schistosomula siRNA control, and another group received 500 schistosomula *SmKI-1* siRNA-electroporated. Forty five days after schistosomula injection, adult worms were perfused from the portal veins, as described previously [64]. Two independent experiments were performed to determine survival rates of *S*. *mansoni*.

### Neutrophil depletion

Neutrophils were depleted by intraperitoneal injection of 50μg of anti-mouse Ly6G (clone 1A8, BioXcell, West Lebanon, NH, USA) 24 hours before infection with 250 schistosomula siRNA control or with 250 schistosomula *SmKI-1* siRNA-electroporated. Forty five days after schistosomula injection, adult worms were perfused from the portal veins, as described above. The neutrophils depletion was maintained with applications of anti-mouse Ly6G antibodies at intervals of 3 days each dose for 12 days. In these experiments, 50 μg of an isotype control antibody (clone LTF-2, BioXcell) was administered as control. Neutrophil depletion was confirmed by flow cytometry analysis of neutrophils or dendritic cells as control present in the spleen of uninfected animals. The populations of neutrophils or dendritic cells were analyzed by staining 1x10^6^ cells for 30 min on 4°C with fluorescent antibodies against Ly6G (PE, clone 1A8, BD Biosciences), CD11b (APC-Cy7, clone M1/70, BD Biosciences), CD11c (FITC, clone HL3, BD Biosciences). Stained cells were acquired in Attune Flow Cytometer (Applied Biosystems, Waltham, MA, USA) and analyzed using FlowJo software (Tree Star, Ashland, OR, USA).

### Schistosomes incubation with neutrophils

*Sm*KI-1 siRNA or control siRNA were delivered to 1-day old schistosomula and resuspended in RPMI medium. Aliquots containing 200 schistosomula were dispensed into flat-bottom, 96-well microtiter plates. In the following day, 10^5^ neutrophils isolated from C57BL/6 mice were added to each well and incubated with schistosomula for 24 hours at 37°C and 7.5% CO_2_. As a viability control, some wells did not receive neutrophils. After incubation time, plates were inspected under an inverted microscope (Olympus Co., Hamburg, Germany). In each well, the percentage of dead schistosomes was scored. Dead schistosomes appear as an opaque and granular appearance, and lacked flame cell activity. This measurement correlated well with a dye-exclusion counting [62].

### Experimental gout arthritis model

Joint inflammation was induced by the intraarticular injection of monosodium urate (MSU) (100 μg per cavity in 10 μL of sterile saline) by inserting a 27.5 G needle through the suprapatellar ligament into the left knee joint cavity. The contralateral knee was injected with the same volume of the vehicle (saline) and used as control. Then one group received intravenously injection of r*Sm*KI-1 (10mg/kg) immediately after MSU administration.

### Nociception assessment

In a quiet room, the mice were placed in acrylic cages (12x10x17 cm high) with a wire grid floor, 15–30 min before the test, for environmental adaptation. In these experiments, an electronic pressure meter was used. It consists of a hand-held force transducer fitted with a polypropylene tip (INSIGHT Instruments, Ribeirão Preto, São Paulo, Brazil) [65]. A non-standard large tip (4.15 mm^2^) was adapted to the probe [66]. An increasing perpendicular force was applied to the central area of the plantar surface of the hind paw to induce the flexion of the knee joint, followed by paw withdrawal. A tilted mirror below the grid provided a clear view of the animal’s hind paw. The end point was characterized by the removal of the paw from the polypropylene tip. After the flexion-elicited withdrawal threshold, the intensity of the pressure was automatically recorded. The value for the response was obtained by averaging two measurements in one hind paw.

### Total and differential synovial cavity cell counts

After induction of joint inflammation, mice were euthanized. Knee synovial cavities were washed twice with 5μL of PBS. Total leukocyte counts were counted using a Neubauer chamber. Differential counts of leucocytes were performed using May-Grunwald-Giemsa-stained cytospin smears (Cytospin 3, ShandonInc.) and the values are reported as the number of cells per cavity.

### Elastase, myeloperoxidase activity and measurement of IL-1β

Supernatant of processed periarticular tissues were used to determinate neutrophil accumulation in periarticular tissues by testing elastase and myeloperoxidase (MPO) activities. Elastase activity was measured using 0.5 mM of the substrate S4760 (Sigma). Leukocyte MPO activity was assessed by measuring the H_2_O_2_-dependent oxidation of TMB. Aliquots of 30 ml were incubated with 120 ml of TMB substrates in 96 well plates. Plates were incubated for 5 min at room temperature and the reaction stopped with H_2_SO_4_ 4%. The optical densities were read at 450 nm in a microwell reader system (mQuant, Bio-Rad, USA). IL-1β was quantified using enzyme-linked immunosorbent assay (ELISA) kit (R&D Systems). All samples were performed in triplicates.

### Infection with *S*. *mansoni* and experimental gout arthritis induction

Briefly, C57BL/6 mice were infected with 50 cercariae from *S*. *mansoni* strain LE by percutaneous exposure of abdominal skin for 1 hour [67]. Experimental gout arthritis was induced 56 days after schistosome infection as described in section “Experimental Gout arthritis Model” and cells were counted as described in section “Total and differential synovial cavity cell counts”.

### Drug-induced liver injury model

Acetaminophen (APAP) (Sigma) was dissolved in warm saline before gavage. Mice were fasted for 15 hours before APAP administration (600 mg/kg) or sterile PBS as vehicle. The animals received intravenously administration r*Sm*KI-1 at a dose of 10 mg/kg 0.5 h before APAP. Mice were then submitted to euthanasia 24 h after APAP injection. Serum alanine aminotransferase (ALT) activity was performed using a kinetic test (Bioclin). Liver fragments were collected for histology (hematoxylin and eosin [H&E]), also for neutrophil elastase and myeloperoxidase activity as described in the preceding section.

### *In vivo* mice imaging

For noninvasive imaging, mice were anesthetized with ketamin and xilasin and injected intravenously with 100 μL of NucRed Dead 647 (Molecular Probes) 5 minutes before imaging at MS FXPRO (Bruker). Liver confocal intravital microscopy was performed as previously described [68]. All fluorophores were injected intravenously 10 minutes before imaging: Sytox Green (100 μL/mouse, 50 μM, Invitrogen) and PE-conjugated anti-GR1 (4μg/mouse; 40μg/mL, eBioscience). Neutrophil counts were performed in the images captured in the confocal microscope.

### Inhibition of neutrophil migration into the pleural cavity by *Sm*KI-1

Carrageenan (2mg/mL), or vehicle (PBS) was injected intrapleurally in a volume of 0.1 ml. After that, mice that received carrageenan were divided into two groups, one group received intravenously injection of r*Sm*KI-1 (10 mg/kg) was termed carrageenan+*Sm*KI-1 group and another that received vehicle only was termed carrageenan group. Mice were sacrificed in a CO_2_ chamber 4 h after the injection. The cells present in the cavity were harvested by injecting 2 mL of PBS and total cell counts were performed in a Neubauer chamber using Turk’s stain. Differential cell counts were performed on cytospin preparations, which were stained with May—Grunwald and Giemsa to identify cell types according to standard morphologic criteria. The results are presented as the number of cells per milliliter.

### Flow cytometry analysis of leukocyte populations

Four hours after carrageenan, or vehicle (PBS) injection, the pleural cells of carrageenan+*Sm*KI-1, carrageenan and control groups were obtained by washing the cavity with PBS. The populations of neutrophils (Ly6G^+^CD11b^+^) macrophages (F4/80^+^CD11b^+^), and T lymphocytes (CD3^+^ cells) were analyzed by staining 2x10^5^ cells for 30 min on ice with fluorescent mAbs against F4/80 (PE, clone A3-1, BioLegend), Ly6G (APC, clone 1A8, BD Biosciences), CD11b (BB515, clone M1/70, BD Biosciences), and CD3 (PE CY5, clone 145-2C11, BD Biosciences). Stained cells were acquired in a BD FACSCanto II (BD Biosciences) and analyzed using FlowJo software (Tree Star, Ashland, OR). The cells were first selected based on size and granularity and CD11b^+^ to separate lymphocyte and macrophage/neutrophil populations. From the CD11b^+^ gate containing the macrophage/neutrophil population, F4/80^+^ cells were selected for macrophages and Ly6G^+^ for neutrophils.

### Thioglycollate induced peritonitis

In order to test a neutrophil-independent inflammation model, C57BL/6 mice received an injection of 3 mL of 3% thioglycollate broth, a non-specific pro-inflammatory stimulus, into their peritoneal cavities. To evaluate the effect of r*Sm*KI-1 on thioglycollate-induced leukocytes influx into the peritoneal cavity, 48 hours after thioglycollate injection, mice were divided in two groups (n = 8) and treated with either rSmKI-1 (10 mg/kg), or vehicle (5 mL/kg of saline). At 72 hours following thioglycollate administration, the animals were sacrificed, the peritoneal leukocyte cells were harvested by lavage of peritoneal cavities with PBS (10 mL) and total cell counts were determined using a Neubauer chamber. Differential counts of leukocytes were performed as described in section “Total and differential synovial cavity cell counts”.

### Statistical analysis

The results from the experimental groups were compared by Student’s *t*-test using the software package GraphPad Prism (La Jolla, CA). Bonferroni adjustments were included for multiple comparisons. The p-values obtained by this method were considered significant if they were p< 0.05, or otherwise stated.

### Accession number

Smp_147730 (*Sm*KI-1) CCD77156.1.

## Supporting information

S1 Fig Biochemical analysis of r*Sm*KI-1, KI Domain and C-terminal. (**A**) *Sm*KI-1 full-length protein sequence. The Kunitz Domain is underlined in black and the canonical segments of the primary and secondary binding loops of SmKI-1 are underlined in red. Glycosylation prediction results show no potential O-glycosylation sites in the sequence (all prediction confidence scores were lower than 0.5), and the single N-glycosylation site that has been annotated for this protein (Asn30, black arrow) is neither in the primary nor in the secondary binding loop. (B) SDS-PAGE stained with Coomassie brilliant blue showing eluted and dialyzed r*Sm*KI-1, KI Domain and c-terminal after purification by Ni_2+_ -charged column chromatography. (C) Western blot analysis of r*Sm*KI-1 and its collect proteins probed with monoclonal mouse anti-His tag antibodies. The molecular weight protein standard (M.W.) is a broad range pre-stained ladder from Fermentas in lane 1; r*Sm*KI-1 in lane 2; KI Domain in lane 3;.C-terminal in lane 4. (D) Average molecular mass and purity of folded KI domain was determined by MALDI-TOF/MS using linear mode on a Bruker instrument AutoFlex III. Precursor charge state M+2H^+^ was detected and the observed average molecular mass was 11,200.0 Da with theoretical average molecular mass of 11,074.0 Da. (E) Circular dichroism analysis (far-UV spectrum) of KI domain showing predominant β-sheet conformation. (F) BHK-21 cells were incubated with r*Sm*KI-1 at the final concentration of 1000, 500, 250, 125, 62.5, 31.25 or 15.62 μg/mL and the cytotoxicity potential was evaluated by MTS assay. The medium alone was used to calculate the percentage of viable cells. The BHK-21 cells were also incubated with a CTAB solution (100 mM) as a positive control of cytotoxicity. *** denotes statistically significant differences (p<0.0001) in relation to cells incubated with medium alone.
(PDF)

S2 Fig *Sm*KI-1 is important in parasite development. (A) Sizes of schistosomula cultured throughout six weeks post siRNA treatment. Sizes of individual schistosomula treated with *Sm*KI-1(white circles) or Control siRNAs (gray squares) are shown. The lines indicate the means for each group. Results shown are representative of two replicate experiments. Significant differences between control group and *Sm*KI-1 group are denoted by *, p< 0.05 or ***, p <0.001. (B) Representative images of schistosomula treated with either control (upper panel) or *Sm*KI-1 (lower panel) siRNAs in all time points evaluated. (C) Number of eggs per gram of liver tissue (mean ± SD) from mice infected with schistosomula treated with control siRNA (black bar) or *Sm*KI-1 siRNA (white bar). (D) Control of viability of schistosomula treated with *Sm*KI-1 siRNA or control siRNA cultured without mouse neutrophils (mean±SD).
(PDF)

S3 Fig Depletion of neutrophils *in vivo* during *Sm*KI-1-suppressed parasites infection. Neutrophils were depleted by treatment with anti-mouse Ly6G and cells were evaluated by flow cytometry, being the percentage of (**a**) neutrophils (Ly6G^+^ CD11b^+^) and (**b**) dendritic cells (CD11c^+^ CD11b^+^), calculated from the total cell numbers. (**c**) Worm burden recovery (mean±SD) of 250 schistosomula siRNA control or 250 schistosomula *SmKI-1* siRNA-electroporated parasites used to infect neutrophil-depleted mice. Results are expressed as the number of worms recovery (mean ± SD) for each treated group (5 mice each). On top of the bars the difference in percentage of worm burden decrease among studied groups.
(PDF)

S4 Fig *Sm*KI-1 does not affect the migration of lymphocytes and macrophages. Forty eight hours after thioglycollate injection (3mL of a 3% solution) into peritoneal cavities, animals received an intravenous dose of *Sm*KI-1 (10 mg/kg) or PBS (vehicle). Twenty-four hours later, we recovered peritoneal cells by washing peritoneal cavities with PBS. Counting of (A) total cells, (B) lymphocytes and (C) macrophages performed by cytospin preparations. ND = not detected.
(PDF)
