# Supplementary figures and images for: Schistosoma mansoni SmKI-1 serine protease inhibitor binds to elastase and impairs neutrophil function and inflammation
Source: PLoS Pathog. 2018 Feb 9;14(2):e1006870. doi: 10.1371/journal.ppat.1006870 (PMC5823468; doi:10.1371/journal.ppat.1006870)

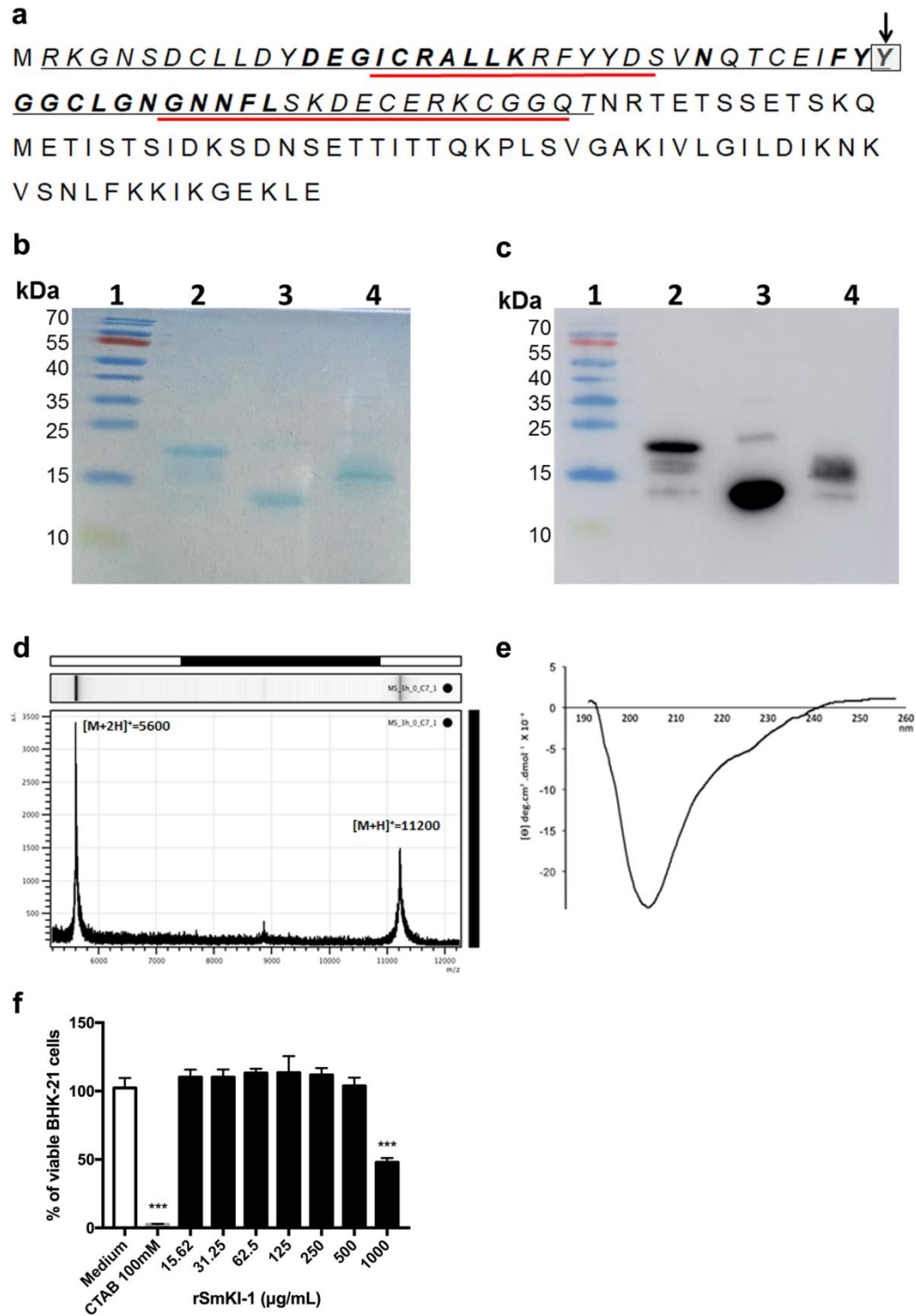

**S1 Fig. Biochemical analysis of rSmKI-1, KI Domain and C-terminal.**

Supplement: S1 Fig — (A) SmKI-1 full-length protein sequence. The Kunitz Domain is underlined in black and the canonical segments of the primary and secondary binding loops of SmKI-1 are underlined in red. Glycosylation prediction results show no potential O-glycosylation sites in the sequence (all prediction confidence scores were lower than 0.5), and the single N-glycosylation site that has been annotated for this protein (Asn30, black arrow) is neither in the primary nor in the secondary binding loop. (B) SDS-PAGE stained with Coomassie brilliant blue showing eluted and dialyzed rSmKI-1, KI Domain and c-terminal after purification by Ni2+ -charged column chromatography. (C) Western blot analysis of rSmKI-1 and its collect proteins probed with monoclonal mouse anti-His tag antibodies. The molecular weight protein standard (M.W.) is a broad range pre-stained ladder from Fermentas in lane 1; rSmKI-1 in lane 2; KI Domain in lane 3;.C-terminal in lane 4. (D) Average molecular mass and purity of folded KI domain was determined by MALDI-TOF/MS using linear mode on a Bruker instrument AutoFlex III. Precursor charge state M+2H+ was detected and the observed average molecular mass was 11,200.0 Da with theoretical average molecular mass of 11,074.0 Da. (E) Circular dichroism analysis (far-UV spectrum) of KI domain showing predominant β-sheet conformation. (F) BHK-21 cells were incubated with rSmKI-1 at the final concentration of 1000, 500, 250, 125, 62.5, 31.25 or 15.62 μg/mL and the cytotoxicity potential was evaluated by MTS assay. The medium alone was used to calculate the percentage of viable cells. The BHK-21 cells were also incubated with a CTAB solution (100 mM) as a positive control of cytotoxicity. *** denotes statistically significant differences (p<0.0001) in relation to cells incubated with medium alone. (PDF) [file ppat.1006870.s001.pdf]

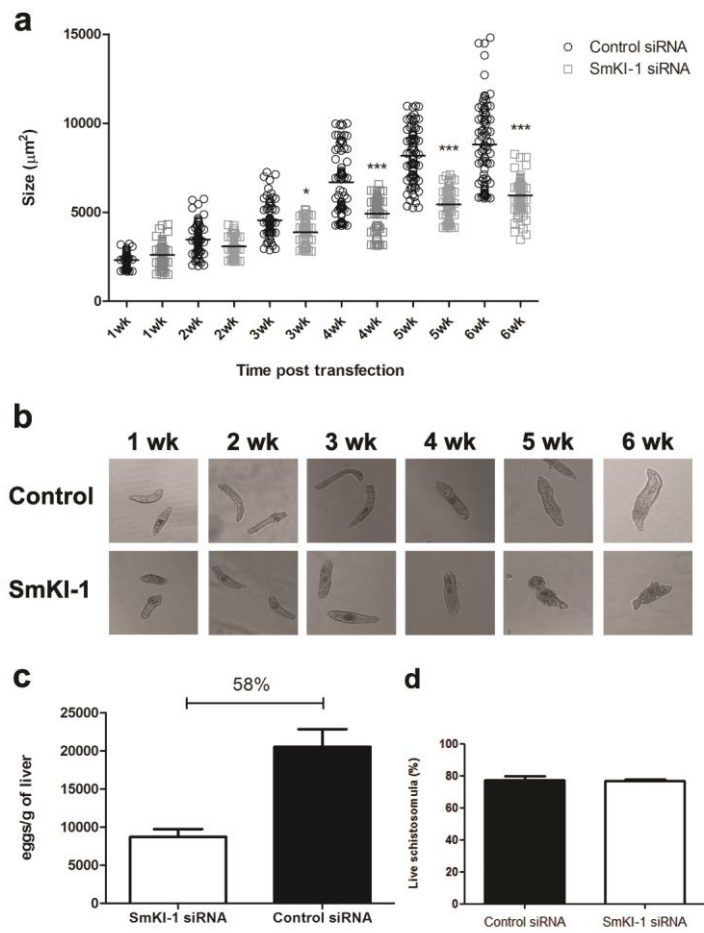

**S2 Fig. *SmKI-1* is important in parasite development.**

Supplement: S2 Fig — (A) Sizes of schistosomula cultured throughout six weeks post siRNA treatment. Sizes of individual schistosomula treated with SmKI-1(white circles) or Control siRNAs (gray squares) are shown. The lines indicate the means for each group. Results shown are representative of two replicate experiments. Significant differences between control group and SmKI-1 group are denoted by *, p< 0.05 or ***, p <0.001. (B) Representative images of schistosomula treated with either control (upper panel) or SmKI-1 (lower panel) siRNAs in all time points evaluated. (C) Number of eggs per gram of liver tissue (mean ± SD) from mice infected with schistosomula treated with control siRNA (black bar) or SmKI-1 siRNA (white bar). (D) Control of viability of schistosomula treated with SmKI-1 siRNA or control siRNA cultured without mouse neutrophils (mean±SD). (PDF) [file ppat.1006870.s002.pdf]

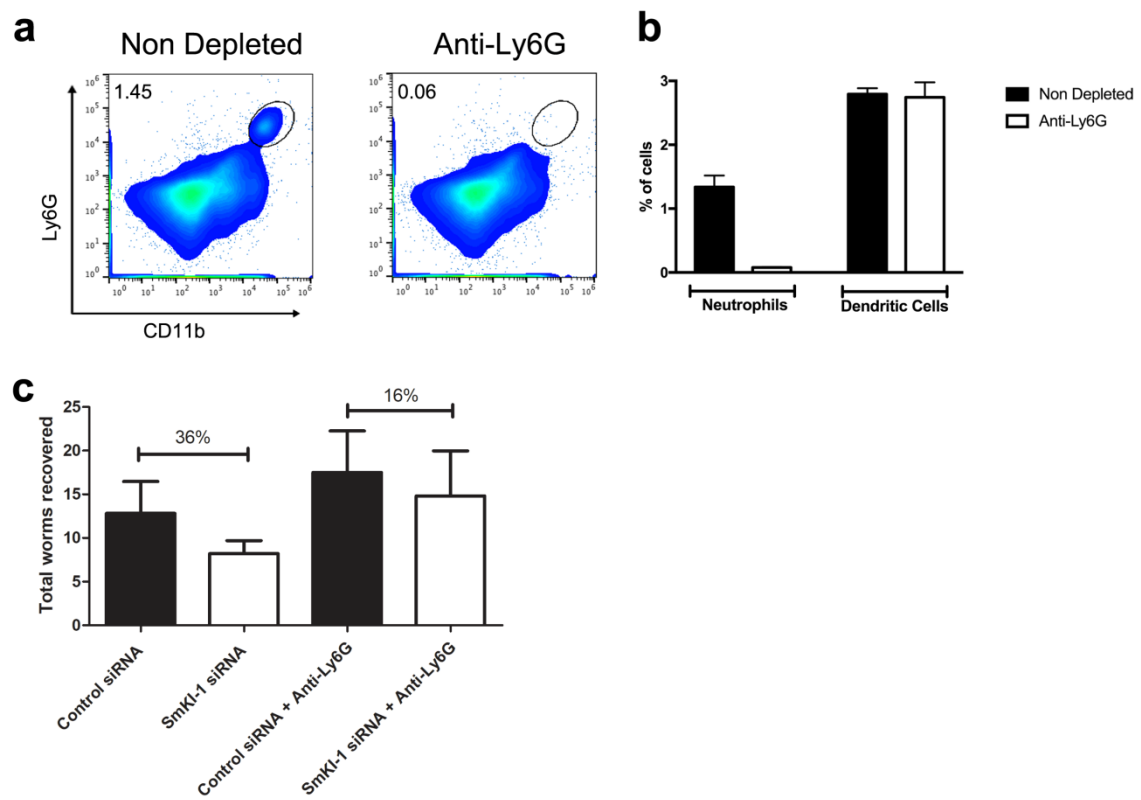

**S3 Fig. Depletion of neutrophils *in vivo* during *SmKI*-1-suppressed parasites infection.**

Supplement: S3 Fig — Neutrophils were depleted by treatment with anti-mouse Ly6G and cells were evaluated by flow cytometry, being the percentage of (a) neutrophils (Ly6G+ CD11b+) and (b) dendritic cells (CD11c+ CD11b+), calculated from the total cell numbers. (c) Worm burden recovery (mean±SD) of 250 schistosomula siRNA control or 250 schistosomula SmKI-1 siRNA-electroporated parasites used to infect neutrophil-depleted mice. Results are expressed as the number of worms recovery (mean ± SD) for each treated group (5 mice each). On top of the bars the difference in percentage of worm burden decrease among studied groups. (PDF) [file ppat.1006870.s003.pdf]

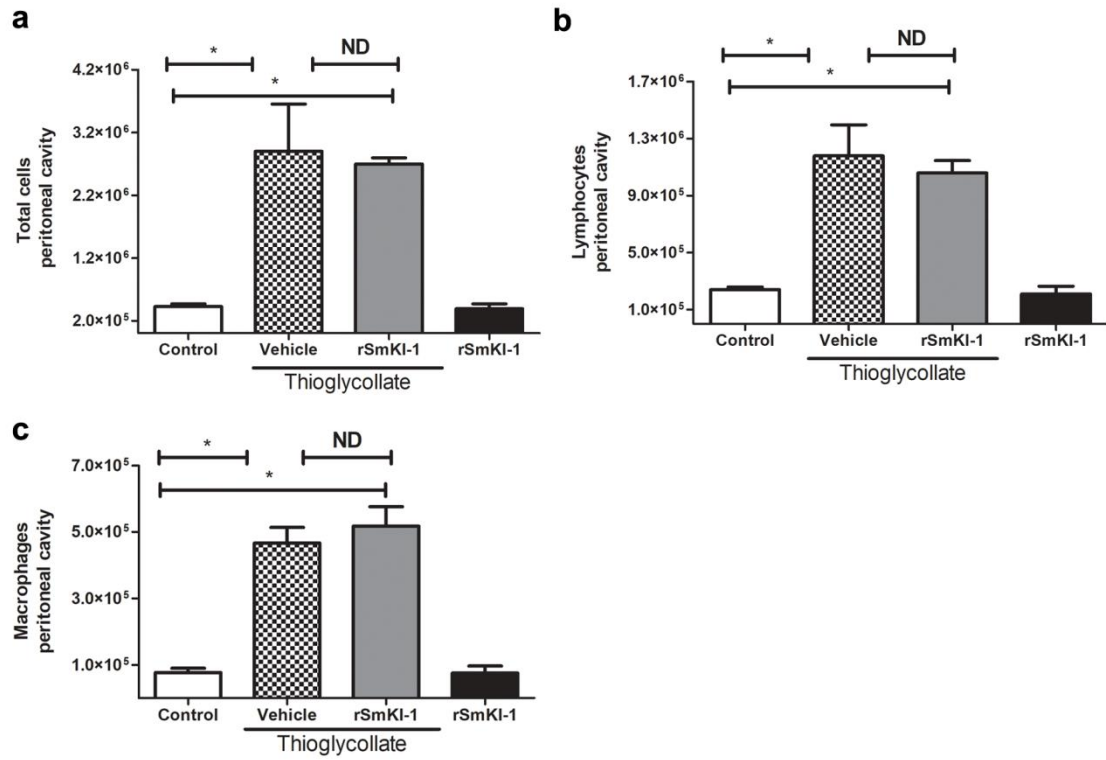

**S4 Fig. *SmKI-1* does not affect the migration of lymphocytes and macrophages.**

Supplement: S4 Fig — Forty eight hours after thioglycollate injection (3mL of a 3% solution) into peritoneal cavities, animals received an intravenous dose of SmKI-1 (10 mg/kg) or PBS (vehicle). Twenty-four hours later, we recovered peritoneal cells by washing peritoneal cavities with PBS. Counting of (A) total cells, (B) lymphocytes and (C) macrophages performed by cytospin preparations. ND = not detected. (PDF) [file ppat.1006870.s004.pdf]
